# ﻿Caribbean Amphipoda (Crustacea) of Panama. Part II: parvorder Hadziidira

**DOI:** 10.3897/zookeys.1195.116721

**Published:** 2024-03-18

**Authors:** Kristine N. White, Sally J. Sir

**Affiliations:** 1 Georgia College & State University, Department of Biological and Environmental Sciences, Aquatic Sciences Center, Milledgeville, GA 31061, USA Georgia College & State University Milledgeville United States of America

**Keywords:** Bocas del Toro, Hadziidae, Hornellidae, identification key, Maeridae, Megaluropidae, Melitidae, Pontogeneiidae

## Abstract

Amphipods in the parvorder Hadziidira are typically associated with algae, sponges, or coral rubble. Members of the parvorder have a gnathopod 2 that is stouter than gnathopod 1, a pair of dorsal robust setae on urosomite 2, and a basofacial robust seta on the uropod 1 peduncle. Within the parvorder, six families are documented from Bocas del Toro, Panama, represented by 26 species. This research documents range extensions for all 26 species and an identification key to the species of Caribbean Hadziidira of Panama is provided.

## Abstract (es)

Los anfípodos del parvorden Hadziidira suelen estar asociados con algas, esponjas o escombros de coral. Los miembros del parvorden tienen un gnatópodo 2 que es más robusto que el gnatópodo 1, un par de setas dorsales robustas en el urosomita 2 y una seta basofacial robusta en el pedúnculo del urópodo 1. Dentro del parvorden se documentan seis familias de Bocas del Toro, Panamá, representadas por 26 especies. Esta investigación documenta extensiones de rango para las 26 especies y se proporciona una clave de identificación para las especies de Hadziidira caribeña de Panamá.

## ﻿Introduction

Parvorder Hadziidira S. Karaman, 1943 is comprised of 1159 species around the world (Lowry and Myers 2013). Members of the parvorder have a gnathopod 2 that is stouter than gnathopod 1, a pair of dorsal robust setae on urosomite 2, and a basofacial robust seta on the uropod 1 peduncle (Lowry and Myers 2013). The parvorder contains 14 families of amphipods: Crangoweckeliidae Lowry & Myers, 2012 (three spp.), Eriopisidae Lowry & Myers, 2013 (87 spp.), Gammaroporeiidae Bousfield, 1979 (one sp.), Hadziidae S. Karaman, 1943 (93 spp.), Maeridae Krapp-Schickel, 2008 (421 spp.), Melitidae Bousfield, 1973 (184 spp.), Metacrangonyctidae Boutin & Messouli, 1988 (20 spp.), Nuuanuidae Lowry & Myers, 2013 (25 spp.), Calliopiidae Sars, 1893 (105 spp.), Cheirocratidae d’Udekem d’Acoz, 2010 (19 spp.), Hornelliidae d’Udekem d’Acoz, 2010 (13 spp.), Megaluropidae Thomas & Barnard, 1986b (16 spp.), Pontogeneiidae Stebbing, 1906 (171 spp.), and Magnovidae Alves, Lowry & Johnson, 2020 (one sp.). Just more than 200 species in the parvorder have been reported from the Caribbean Sea, representing ten families, but none of the species have been previously reported from Caribbean Panama (LeCroy et al. 2009; Martín et al. 2013).

Within the parvorder Hadziidira, 26 species of amphipods were collected from Bocas del Toro, Panama, with representatives from families Hadziidae, Hornellidae, Maeridae, Megaluropidae, Melitidae, and Pontogeneiidae. All species are diagnosed herein. An identification key is provided to distinguish between the Hadziidira species known from the Caribbean waters of Panama.

## ﻿Materials and methods

Various substrates were collected by hand and placed into buckets or plastic bags from various sites around Bocas del Toro, Panama at depths of 0.2–12 m. Coral rubble was elutriated with freshwater to remove amphipods and other samples were sorted through by hand. Live amphipods were sorted to morphospecies, placed in clove oil for imaging, and preserved in 99.5% EtOH for later examination. Preserved specimens were transferred to glycerol, measured from the tip of the rostrum to the base of the telson, and dissected under a stereomicroscope. Specimens were illustrated using a Meiji MT5900L phase contrast microscope with an Olympus U-DA drawing tube attached or an Olympus BH2 differential interference contrast microscope with an Olympus BH2-DA drawing tube attached. Illustrations were digitally inked following Coleman (2003) in Adobe Illustrator 2020 using a Wacom^®^ Intuos Pro Pen Tablet. Specimens are deposited in the Smithsonian Institution, U.S. National Museum of Natural History (**USNM**) and the Gulf Coast Research Laboratory Museum (**GCRL**).

## ﻿Results

**Parvorder Hadziidira S. Karaman, 1943**

### ﻿Superfamily Hadzoidea S. Karaman, 1943

#### ﻿Family Hadziidae S. Karaman, 1943

##### ﻿Genus Dulzura

Taxon classificationAnimaliaAmphipodaHadziidae

J.L. Barnard, 1969

938188E3-A6FB-504C-B773-C2570423BEA7

###### Diagnosis.

Antenna 1 accessory flagellum 2-articulate; lower lip lacking inner lobes; gnathopod 1 smaller than gnathopod 2; coxa 4 not excavate posteriorly; pleonites 1–3 smooth; uropod 3 inner ramus minute, outer ramus greatly elongated, 2-articulate, article 2 short; telson deeply cleft.

##### ﻿ Dulzura schoenerae

Taxon classificationAnimaliaAmphipodaHadziidae

(Fox, 1973)

767EE483-64F2-51B9-B62F-F7B45F0AF060

Figs 1 , 27A

Eriopisa schoenerae Fox, 1973: 153–159, figs 5–8.
Protohadzia schoenerae : Zimmerman and Barnard 1977: 571–579, figs 1–5; Thomas 1993: 45, figs 48, 58; LeCroy 2000: 69, fig. 101.

###### Material examined.

Panama • 2.8–5 mm • 1 ♀; Bocas del Toro, Hospital Bight; 9.304483°N, 82.131617°W; depth 1.5 m, surface of dead coral; 7 Aug 2005; S.E. LeCroy leg.; GCRL 6627 • 2 ♂, 1 ♀; Bocas del Toro, Isla Solarte; 9.244333°N, 82.250733°W; depth 0.5 m, *Halimeda* and *Thalassia*; 9 Aug 2005; S.E. LeCroy leg.; GCRL 6628 • 5 ♀; Bocas del Toro, Isla Solarte; 9.290110°N, 82.189732°W; depth 1–5 m, among coral rubble; 8 Aug 2021; K.N. White leg.; USNM 1703494.

###### Diagnosis.

Male gnathopod 2 propodus palm with acute apical protrusion, much larger than female gnathopod 2; epimeron 3 with simple small posteroventral tooth; telson apically acute with apical spines.

###### Distribution.

USA: Florida from Biscayne Bay to the Dry Tortugas (Thomas 1993); Bahama Islands: Bimini (Fox 1973); Puerto Rico: La Parguera (Zimmerman and Barnard 1977); Cuba: Caleta de San Lázaro (Ortiz et al. 2001); Mexico: Puerto Morelos National Park (Winfield et al. 2023); Panama: Bocas del Toro (present study).

###### Ecology and remarks.

These amphipods are associated with coral rubble, algae, and seagrass near coral reefs at depths of 1–5 m. Panamanian specimens agree closely with previous descriptions of the species and are easily distinguishable based on the uropod 3, gnathopod 2, and epimeron 3, even in smaller specimens.

**Figure 1.**
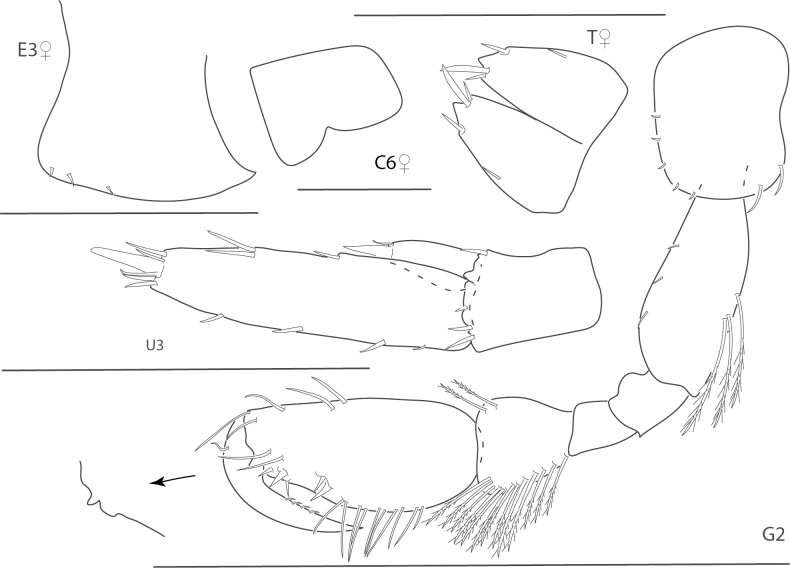
*Dulzuraschoenerae*, female, 2.8 mm, epimeron 3, coxa 6, telson, uropod 3, gnathopod 2 medial. Scale bars: 0.5 mm.

#### ﻿Family Maeridae Krapp-Schickel, 2008

##### ﻿Genus Ceradocus

Taxon classificationAnimaliaAmphipodaMaeridae

Costa, 1853

198EAFC2-59CD-5506-A9A9-F857777FE081

###### Diagnosis.

Eyes oval. Maxilla 2 inner plate with dense facial setae. Coxa 1 produced anteroventrally. Pereopod 7 basis weakly expanded, posterior margin serrate. Urosomite segments usually serrate. Uropod 3 rami foliaceous, apically truncate, extending beyond tips of uropods 1 and 2. Telson deeply cleft.

##### ﻿ Ceradocus sheardi

Taxon classificationAnimaliaAmphipodaMaeridae

Shoemaker, 1948

DB3369FA-0FCD-549B-B50D-388BED18D17F

Figs 2 , 27B

Ceradocus sheardi Shoemaker, 1948: 7–9, fig. 2; Thomas 1993: 43, figs 51, 56; LeCroy 2000: 73, fig. 124.

###### Material examined.

Panama • 5–10 mm • 1 ♀; Bocas del Toro, Swan Cay; 9.4536°N, 82.300033°W; depth 2 m, coral rubble; 24 June 2023; K.N. White leg.; USNM 1703495 • 1 ♂; Bocas del Toro, Cayo Zapatilla 1; 9.269967°N, 82.0587°W; depth 10–11 m, coral rubble; 28 June 2023; K.N. White leg.; USNM 1703496.

###### Diagnosis.

Antenna 1 accessory flagellum 7- or 8-articulate. Gnathopod 2 not sexually dimorphic, right and left sides similar; propodus enlarged, palm convex, defined by notch. Pereopod 7 basis without posterodistal lobe. Pleosome and urosome dorsally serrate. Epimera 1–3 posterior margins serrate. Telson approximately as long as wide with strong apical spines.

**Figure 2.**
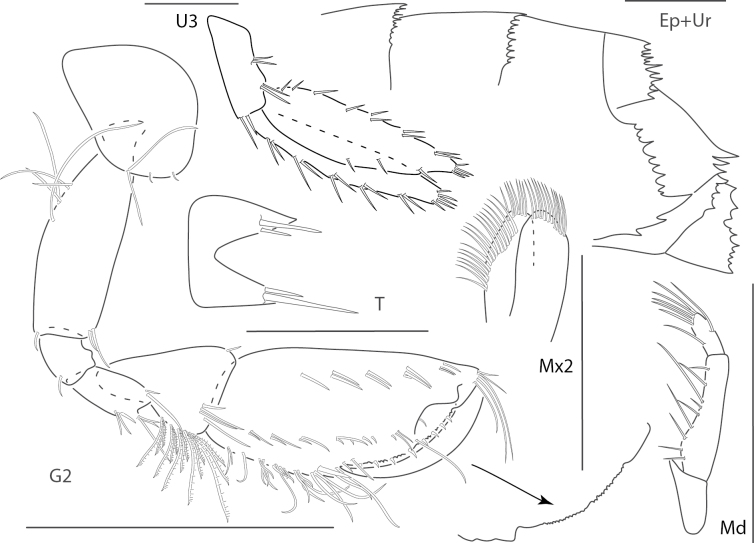
*Ceradocussheardi*, male, 5.2 mm, gnathopod 2 medial, gnathopod 2 palm with setae removed, telson, epimeron and urosome uropod 3, maxilla 2, mandibular palp. Scale bars: 0.5 mm.

###### Distribution.

USA: South Florida from Biscayne Bay to the Dry Tortugas (Thomas 1993); Puerto Rico (LeCroy 2000); Cuba (Shoemaker 1948; Varela et al. 2003); Mexico: Yucatan (Shoemaker 1948; Thomas 1993); Belize (Thomas 1993); Panama: Bocas del Toro (present study).

###### Ecology and remarks.

These amphipods are common among coral rubble and under rocks at depths of 1–52 m. Panamanian specimens agree closely with previous descriptions of the species and are easily distinguishable based on the uropod 3, gnathopod 2, and heavily serrate epimeron and urosome.

##### ﻿ Ceradocus shoemakeri

Taxon classificationAnimaliaAmphipodaMaeridae

Fox, 1973

516F52C8-55A4-548D-A93B-3B67D60681A9

Figs 3 , 27C

Ceradocus shoemakeri Fox, 1973: 147–152, figs 1–4; LeCroy 2000: 73, fig. 121.

###### Material examined.

Panama • 2.2–7 mm • 1 ♀; Bocas del Toro, Hospital Point; 9.3336°N, 82.218833°W; depth 15- m, coral rubble and *Halimeda*; 6 Aug 2005; S. DeGrave leg.; GCRL 6629 • 1 ♀; Bocas del Toro, San Cristobal; 9.284977°N, 82.294533°W; depth 1–3 m, *Halimeda*; 21 June 2023; K.N. White leg.; USNM 1703497 • 1 ♂, 2 ♀; Cayo Zapatilla 1; 9.269967°N, 82.0587°W; depth 10–11 m, coral rubble; 28 June 2023; K.N. White leg.; USNM 1703498.

###### Diagnosis.

Antenna 1 accessory flagellum 4- or 5-articulate. Gnathopod 2 sexually dimorphic, right and left sides dissimilar; enlarged side palm oblique with two subquadrate and one triangular projection. Pereopod 7 basis with small posterodistal lobe. Pleosome and urosome dorsally smooth. Epimera 1–3 posterior margins serrate. Telson approximately as long as wide with subapical setae.

**Figure 3.**
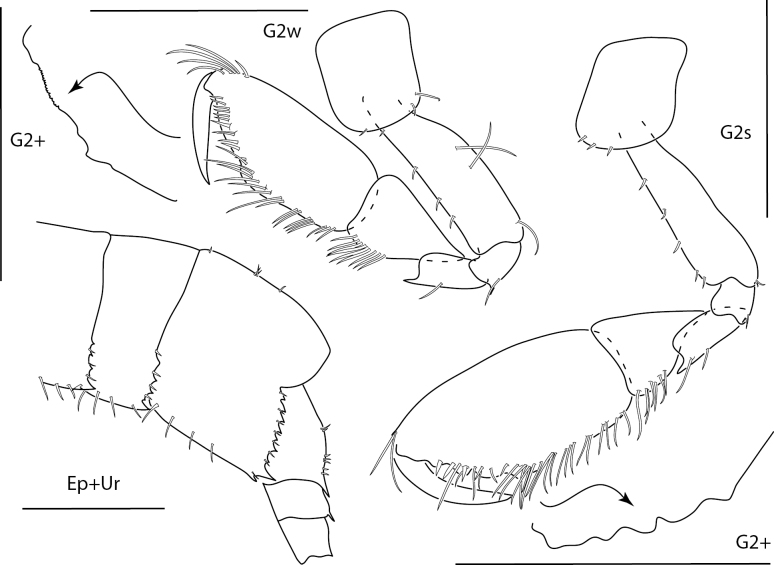
*Ceradocusshoemakeri*, male, 2.2 mm, gnathopod 2 weak side lateral, weak gnathopod 2 propodus palm enlarged with setae removed, gnathopod 2 strong side lateral, strong gnathopod 2 propodus palm enlarged with setae removed, epimeron and urosome. Scale bars: 0.5 mm.

###### Distribution.

U.S.A.: South Florida from Biscayne Bay to the Dry Tortugas; Apalachee Bay, Perdido Key, Florida (LeCroy 2000); Bahama Islands: Bimini (Fox 1973); Panama: Bocas del Toro (present study).

###### Ecology and remarks.

These amphipods are associated with coral rubble, algae, and sponges at depths of 0.5–15 m. Panamanian specimens have a dorsally smooth pleosome and urosome; Fox (1973) mentioned that occasionally there is a mid-dorsal posterior tooth on pleonites 4 and/or 5.

##### ﻿Genus Elasmopus

Taxon classificationAnimaliaAmphipodaMaeridae

Costa, 1853

D4AB30DE-997B-5535-A9F7-95685603BD83

###### Diagnosis.

Antenna 1 elongate, at least 1/3 of body length; accessory flagellum 2- or 3- articulate. Mandibular palp article 3 falcate, anterior margin with pectinate setae. Uropod 1 peduncle with basofacial spine; uropod 3 rami subequal in length.

##### ﻿ Elasmopus balkomanus

Taxon classificationAnimaliaAmphipodaMaeridae

Thomas & Barnard, 1988

AF169F01-85A1-5153-AF19-A3586CB02A15

Figs 4 , 27D

Elasmopus balkomanus Thomas & Barnard, 1988: 838–842, figs 1–3; LeCroy 2000: 86, fig. 133.

###### Material examined.

Panama • 1.6–8 mm • 1 ♀; Bocas del Toro, Crawl Cay; 9.2475°N, 82.1290°W; depth 5 m, among coral rubble; 12 Aug 2021; K.N. White leg.; USNM 1703499 • 1 ♂; Bocas del Toro, Crawl Cay; 9.245967°N, 82.136867°W; depth 1–4 m, among sand; 25 June 2023; K.N. White leg.; USNM 1703500.

###### Diagnosis.

Gnathopod 1 male propodus subovate, palm oblique, female propodus slender, ventral margin straight. Gnathopod 2 male propodus palm densely setose with crenulate longitudinal ridge on medial surface, female propodus with 2 spines at palmar angle. Pereopod 5 basis posterior margin concave. Pereopod 7 basis posterior margin without long setae, articles 4 and 5 of male unexpanded, slender. Epimeron 3 posteroventral margin with small tooth. Telson inner lobes shorter than outer lobes, apically acute.

###### Distribution.

U.S.A.: South Florida, Looe Key (Thomas and Barnard 1988; Thomas 1993), Biscayne Bay (LeCroy 2000); Panama: Bocas del Toro (present study).

###### Ecology and remarks.

These amphipods are associated with algal turf and coral rubble at depths of 1–5 m. Panamanian specimens have a less setose gnathopod 2 propodus and less concave pereopod 5 basis than described by Thomas and Barnard (1988). The gnathopod 2 propodus is also less inflated than previously described. This suggests variation in these characters with size as the Panamanian specimens were smaller than those described by Thomas and Barnard (1988). Other characters agree with previous descriptions of this species.

**Figure 4.**
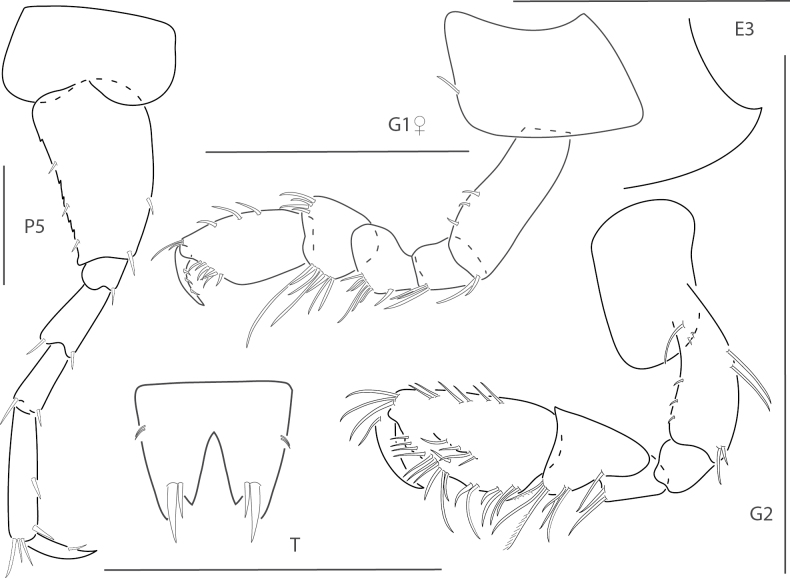
*Elasmopusbalkomanus*, male 1.6 mm, pereopod 5, telson, gnathopod 2 medial, epimeron 3 posterovental corner; female, 3.7 mm, gnathopod 1 lateral. Scale bars: 0.5 mm.

##### ﻿ Elasmopus elieri

Taxon classificationAnimaliaAmphipodaMaeridae

Ortiz, Lalana & Varela, 2004

4814367F-1C33-5458-873A-E35AE08D4952

Figs 5 , 27E

Elasmopus elieri Ortiz, Lalana & Varela, 2004: 36–39, figs 1, 2.

###### Material examined.

Panama • 3.5–6 mm • 9 ♂, 9 ♀; Bocas del Toro, Lime Point; 9.4149°N, 82.33225°W; depth 0.2–0.5 m, among red algae and coral rubble; 5 Aug 2005; S. DeGrave leg.; GCRL 6630.

###### Diagnosis.

Gnathopod 1 propodus subrectangular, palm oblique. Gnathopod 2 male propodus elongate, palm concave, with large distal triangular tooth, medial surface with two subtriangular processes; female propodus elongate, narrow, palm oblique with two spines at palmar angle. Pereopod 5 basis posterior margin evenly convex. Pereopod 7 basis posterior margin without long setae. Epimeron 3 posteroventral margin with small tooth. Telson inner lobes subequal in length with outer lobes, apically rounded.

###### Distribution.

Cuba: Cayo Diego Pérez (Ortiz et al. 2004); Panama: Bocas del Toro (present study).

###### Ecology and remarks.

These amphipods are associated with algae, and coral rubble at depths of 0–3 m. Panamanian specimens closely resemble specimens described by Ortiz et al. (2004) and can be readily distinguished from other species based on the unique shape of the gnathopod 2 propodus in males.

**Figure 5.**
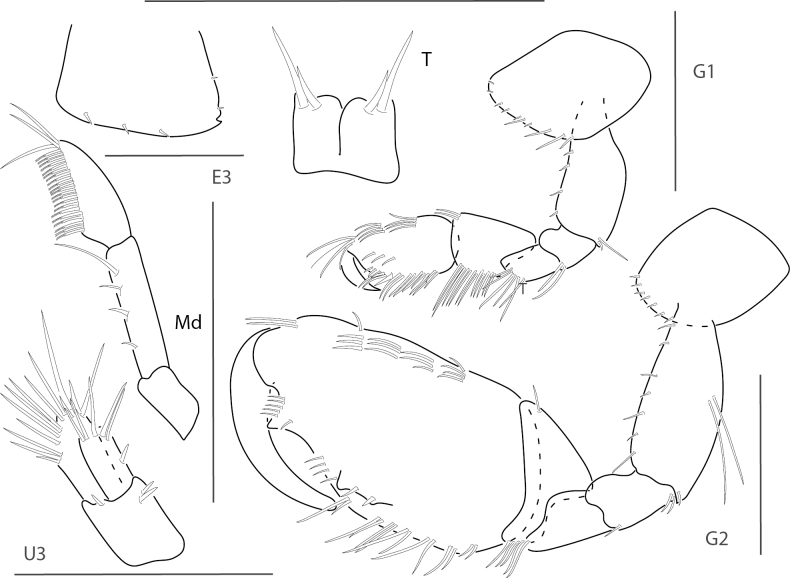
*Elasmopuselieri*, male, 4.2 mm, epimeron 3, telson, gnathopods 1 and 2 medial, manidibular palp, uropod 3. Scale bars: 0.5 mm.

##### ﻿ Elasmopus levis

Taxon classificationAnimaliaAmphipodaMaeridae

(Smith, 1873)

089F1316-1C8E-5998-B9A6-B5C9852D9F24

Figs 6 , 27F

Maera levis Smith, 1873: 559.
Elasmopus laevis : LeCroy 2000: 87, fig. 137.

###### Material examined.

Panama • 5–10 mm • 1 ♂; Bocas del Toro, Playa Bluff; 9.3905°N, 82.23725°W; depth 0 m, among algae; 5 Aug 2005; T.A. Haney leg.; GCRL 6631 • 1 ♀; Bocas del Toro, Hospital Point; 9.3336°N, 82.218883°W; depth 15 m, among coral rubble; 6 Aug 2005; S. DeGrave leg.; GCRL 6632 • 2 ♀, 2 ♂, 3 juvenile; Pigeon Key Reef; depth 0.5–1 m, among *Halimeda*; 9 Aug 2005; T.A. Haney leg.; GCRL 6633 • 1 ♂; Bocas del Toro, STRI Point; 9.34872°N, 82.26258°W; depth 12 m, among coral rubble; 6 Aug 2021, K.N. White leg.; USNM 1703501 • 1 ♀, 1 ♂; Bocas del Toro, Drago; 9.418056°N, 82.3375°W; depth 2–3 m, among coral rubble, 9 Aug 2021; K.N. White leg.; USNM 1703502 • 2 ♀; Bocas del Toro, Cayo Zapatilla 1; 9.269967°N, 82.0587°W; depth 10–11 m, among coral rubble; 28 June 2023; K.N. White leg.; USNM 1703503.

###### Diagnosis.

Gnathopod 1 propodus subrectangular, palm transverse. Gnathopod 2 male propodus with deep medial depression, lined with groups of long setae along ventral margin, with one small medial tooth, female propodus with two spines at palmar angle slightly longer than spines on palmar margin. Pereopod 5 basis posterior margin evenly convex. Pereopod 7 basis posterior margin without long setae, articles 4 and 5 of male expanded, articles 5 and 6 with long posterior setae. Epimeron 3 posteroventral margin with small tooth, sometimes with serrated edge. Telson inner lobes longer than outer lobes, apically subacute.

###### Distribution.

U.S.A.: Cape Cod, Massachusetts (Bousfield 1973) to South Florida (LeCroy 2000); Mexico: Yucatan (McKinney 1977); Panama: Bocas del Toro (present study).

###### Ecology and remarks.

These amphipods are associated with seagrass, algae, and coral rubble at depths of 0–11 m. Panamanian specimens show variation in the level of acuteness of the telson apices and the posterior margin of epimeron 3. Gulf of Mexico and western Atlantic specimens are described as having acute telson lobes (subacute in Panamanian specimens) and all Panamanian specimens show the posteroventral tooth on epimeron 3, whereas this species is sometimes described as having an entire epimeron 3.

**Figure 6.**
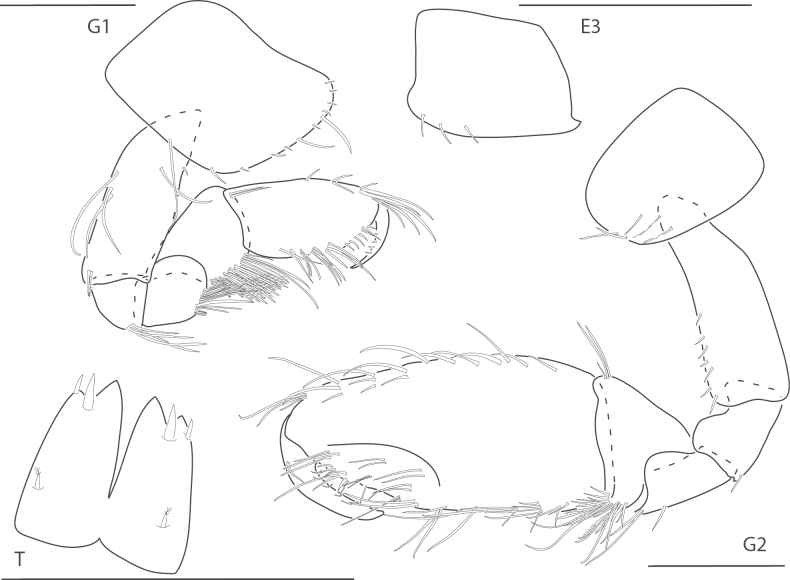
*Elasmopuslevis*, male, 4.2 mm, gnathopod 1 lateral, gnathopod 2 medial, epimeron 3, telson. Scale bars: 0.5 mm.

##### ﻿ Elasmopus longipropodus

Taxon classificationAnimaliaAmphipodaMaeridae

Senna & Souza-Filho, 2011

4B746FBF-1B5E-592E-9198-949EEBA91356

Figs 7 , 27G

Elasmopus rapax (non Elasmopusrapax Costa, 1851): Soares et al. 1987/89: 244, pl. 3, figs 1–12; Wakabara et al. 1991: 73.
Elasmopus aff. rapax : Souza-Filho and Senna 2009: 67.
Elasmopus longipropodus Senna & Souza-Filho, 2011: 59–66, figs 1–6.

###### Material examined.

Panama • 2.5–7 mm • 3 ♂, 3 ♀; Bocas del Toro, Swan Cay; 9.453333°N, 82.298333°W; depth 2–3 m, among algae; 4 Aug 2005; S. DeGrave leg.; GCRL 6634 • 2 ♂, 5 ♀, 2 juvenile; Bocas del Toro, Hospital Point; 9.3336°N, 82.218883°W; depth 15 m, among coral rubble; 6 Aug 2005; S. DeGrave leg.; GCRL 6635 • 2 ♂, 7 ♀, 2 juvenile; Bocas del Toro, Isla Solarte channel; 9.294574°N, 82.173114°W; depth 2 m, among *Halimeda*, 8 Aug 2021; K.N. White leg.; USNM 1703504 • 1 ♀, 3 ♂; Bocas del Toro, San Cristobal; 9.2625°N, 82.235°W; depth 15 m, among coral rubble, 10 Aug 2021; K.N. White leg.; USNM 1703505 • 1 ♂, 2 ♀, 1 juvenile; Bocas del Toro, Crawl Cay; 9.2376°N, 82.1438°W; depth 1.5–3 m, among *Halimeda*, 11 Aug 2021; K.N. White leg.; USNM1703506 • 1 ♂, 2 ♀; Bocas del Toro, Hospital Point; 9.331967°N, 82.214817°W; depth 1–3 m, among *Halimeda*, 22 Jun 2023; K.N. White leg.; USNM1703507 • 4 ♂, 1 ♀; Bocas del Toro, Swan Cay; 9.4536°N, 82.300033°W; depth 1–4 m, among red algae, 24 Jun 2023; K.N. White leg.; USNM 1703508 • 1 ♂,1 juvenile, Bocas del Toro, Crawl Cay; 9.245967°N, 82.136867°W; depth 1–4 m, among green algae; 25 June 2023; K.N. White leg.; USNM 1703509 • 2 ♂; Bocas del Toro, Crawl Cay; 9.24756°N, 82.12901°W; depth 5–8 m, among coral rubble, 26 Jun 2023; K.N. White leg.; USNM 1703510 • 1 ♂, 8 ♀, 1 juvenile; Bocas del Toro, Hospital Point; 9.333383°N, 82.218467°W; depth 11 m, among coral rubble, 26 Jun 2023; K.N. White leg.; USNM 1703511.

###### Diagnosis.

Gnathopod 1 propodus subovate, palm oblique. Gnathopod 2 propodus elongate, male palm shorter than posterior margin with two large, rounded processes and one large subacute process at palmar angle. Pereopod 7 basis posterior margin with long setae, articles 4 and 5 of male unexpanded. Epimeron 3 posterior margin serrate. Uropod 3 rami subequal or slightly unequal in length. Telson inner lobes longer than outer lobes, apically rounded.

###### Distribution.

Brazil: from Rio Grande do Norte State to Rio de Janeiro State (Senna and Souza-Filho, 2011); Panama: Bocas del Toro (present study).

###### Ecology and remarks.

These amphipods are associated with algae, sponges, and coral rubble at depths of 1.5–15 m. Panamanian specimens agree closely with the description provided by Senna and Souza-Filho (2011) and can be readily distinguished from other species by the shape of the gnathopod 2 propodus.

**Figure 7.**
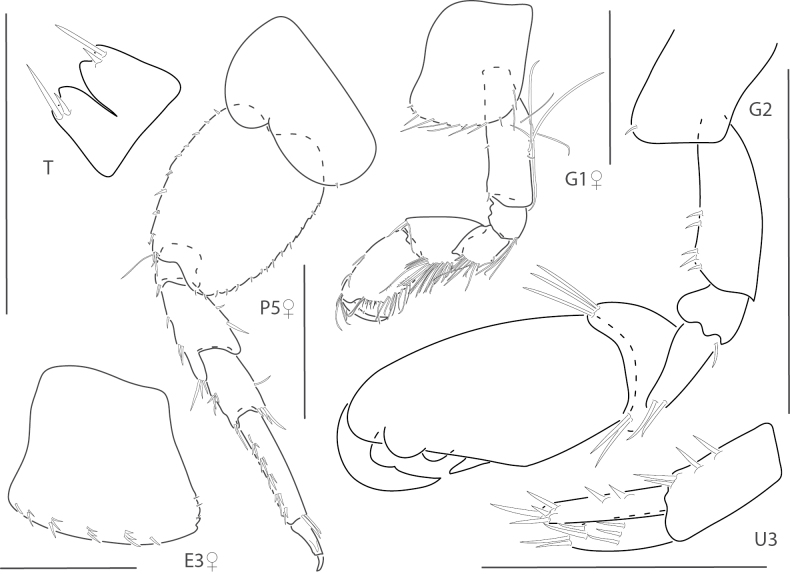
*Elasmopuslongipropodus*, female, 2.5 mm, epimeron 3, pereopod 5, gnathopod 1 lateral; male, 4.5 mm, telson, gnathopod 2 lateral, uropod 3. Scale bars: 0.5 mm.

##### ﻿ Elasmopus pocillimanus

Taxon classificationAnimaliaAmphipodaMaeridae

(Bate, 1862)

C85447C4-3C6A-5147-BB36-91ECB06E7EC5

Figs 8 , 28A

Maera pocillimanus Bate, 1862: 191, pl. 34, fig. 7.
Elasmopus pocillimanus : Della Valle 1893: 733, pl. 1, fig. 4, pl. 22, figs 23–25; LeCroy 2000: 89, fig. 138.

###### Material examined.

Panama • 4–8 mm • 1 ♀; Bocas del Toro, Isla Solarte; 9.2475°N, 82.1290°W; depth 1–4 m, among coral rubble; 8 Aug 2021; K.N. White leg.; USNM 1703512 • 1 ♂; Bocas del Toro, Drago; 9.418056°N, 82.3375°W; depth 2–4 m, among coral rubble; 9 Aug 2021; K.N. White leg.; USNM 1703513 • 1 ♀; Bocas del Toro, Hospital Point; 9.331967°N, 82.214817°W; depth 1–3 m, among coral rubble, 22 Jun 2023; K.N. White leg.; USNM 1703514 • 1 ♂; Bocas del Toro, Swan Cay; 9.4536°N, 82.300033°W; depth 1–4 m, among coral rubble, 24 Jun 2023; K.N. White leg.; USNM 1703515.

###### Diagnosis.

Gnathopod 1 propodus subrectangular, palm transverse. Gnathopod 2 male propodus with deep medial depression, lined with groups of long setae along ventral margin, with one small medial tooth, female propodus with two spines at palmar angle slightly longer than spines on palmar margin. Pereopod 5, basis posterior margin evenly convex. Pereopod 7 basis posteroventral margin with long setae, articles 4 and 5 of male expanded, articles 5 and 6 with long posterior setae. Epimeron 3 posteroventral margin entire, sometimes with small tooth or weakly crenulate. Telson inner lobes longer than outer lobes, apically rounded.

###### Distribution.

Cosmopolitan distribution in warm temperate and tropical waters (McKinney 1977; Karaman 1982; Thomas 1993) most likely refers to multiple species. U.S.A.: New England through Gulf of Mexico and Caribbean (LeCroy 2000); Panama: Bocas del Toro (present study).

###### Ecology and remarks.

These amphipods are associated with algae and coral rubble at depths of 0–30 m. Panamanian specimens show variation in the amount of crenulation of epimeron 3 posterior margin, but consistently have rounded apices on the telson. Other characters align well with previous descriptions of *E.pocillimanus*. See LeCroy (2000) for a discussion of the status of the species.

**Figure 8.**
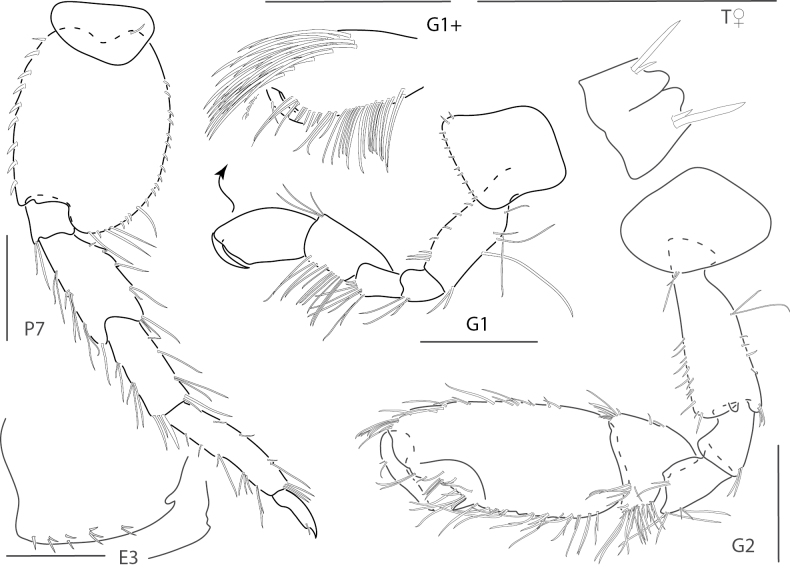
*Elasmopuspocillimanus*, female, 5.0 mm, telson; male, 6.1 mm, pereopod 7, gnathopod 1 lateral, setae removed from propodus, gnathopod 1 propodus medial enlarged, epimeron 3 (two variations), gnathopod 2 medial. Scale bars: 0.5 mm.

##### ﻿ Elasmopus thomasi

Taxon classificationAnimaliaAmphipodaMaeridae

Ortiz & Lalana, 1994

4D06D3A5-5141-5815-8421-E84C82A45684

Figs 9 , 28B

Elasmopus thomasi Ortiz & Lalana, 1994: 297–301, figs 4–6.

###### Material examined.

Panama • 2–5 mm • 2 ♀, 4 juvenile.; Bocas del Toro, Swan Cay; 9.453333°N, 82.298333°W; depth 3 m, among algae; 4 Aug 2005; T.A. Haney leg.; GCRL 6636 • 1 ♂; Bocas del Toro tah2005.001; Aug 2005; T.A. Haney leg.; GCRL 6638 • 2 ♂, 3 ♀; Bocas del Toro, Mangrove Inn; depth 1–1.5 m, among *Halimeda*; 3 Aug 2005; M. Faust leg.; GCRL 6637 • 1 ♂, 1 ♀; Bocas del Toro, San Cristobal; 9.284977°N, 82.294533°W; depth 1–3 m, among sponges; 21 Jun 2023; K.N. White leg.; USNM 1703516 • 4 ♀, 1 ♂; Bocas del Toro, Hospital Point; 9.331967°N, 82.214817°W; depth 1–3 m, among sand and coral rubble; 22 Jun 2023; K.N. White leg.; USNM 1703517.

###### Diagnosis.

Gnathopod 1 propodus subovate, palm oblique. Gnathopod 2 propodus subovate, male palm with three processes and two notches; dactylus resting in notch at palmar angle. Pereopod 5 basis posterior margin evenly convex. Pereopod 7 basis posterior margin without long setae, articles 4 and 5 of male unexpanded. Epimeron 3 posterior margin serrate. Uropod 3 inner ramus shorter than outer ramus. Telson inner lobes longer than outer lobes, apically rounded.

###### Distribution.

Cuba: North coast (Ortiz and Lalana 1994); Panama: Bocas del Toro (present study).

###### Ecology and remarks.

These amphipods are associated with algae and coral rubble at depths of 0–3 m. Panamanian specimens closely resemble specimens described from Cuba and can be readily distinguished by the shape of gnathopod 2 propodus and the rounded apices of the telson.

**Figure 9.**
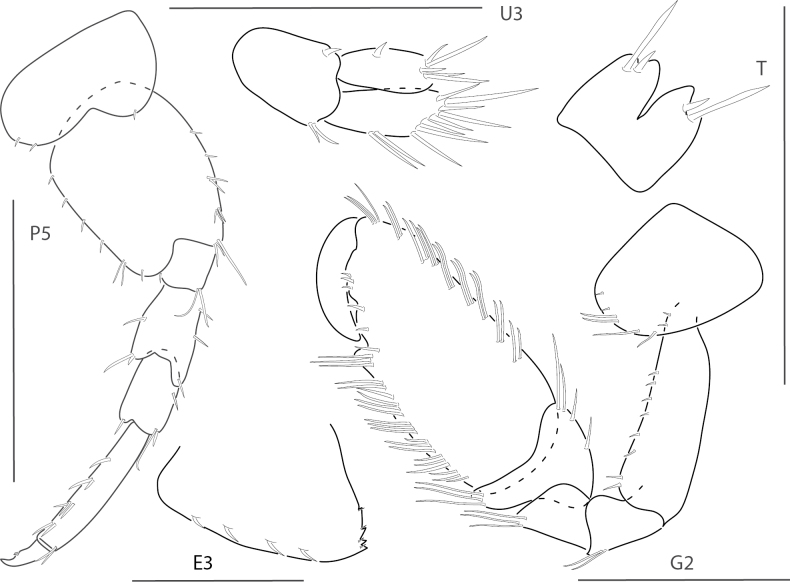
*Elasmopusthomasi*, male, 3.0 mm, pereopod 5, uropod 3, telson, gnathopod 2 medial, epimeron 3. Scale bars: 0.5 mm.

##### ﻿Genus Meximaera

Taxon classificationAnimaliaAmphipodaMaeridae

J.L. Barnard, 1969

154D66ED-BAE7-5321-8A5F-4566F06092A2

###### Diagnosis.

Antenna 1 accessory flagellum 4-articulate. Mandibular palp article 2 longer than articles 1 or 3, article 3 slender, linear. Lower lip inner lobes present. Maxilla 1 and 2 inner plates lacking or with scarce medial setae. Gnathopods 1 and 2 small, subchelate. Epimeral plates smooth. Uropod 3 rami subequal in length, outer ramus minutely bi-articulate. Telson cleft, lobes apically excavated.

##### ﻿ Meximaera diffidentia

Taxon classificationAnimaliaAmphipodaMaeridae

J.L. Barnard, 1969

CE3F6A04-48CA-54F8-BA7D-214A243D1B5C

Figs 10 , 28C

Meximaera diffidentia Barnard, 1969b: 209–210, figs 21–22; Krapp-Schickel and Vader 2009: 2082–2085, fig. 10.
Maera caroliniana : Bynum and Fox 1977: 11–14, figs 6, 7; LeCroy 2000: 99, fig. 143.

###### Material examined.

Panama • 4–6 mm • 1 ♂; Bocas del Toro, STRI Point; among coral rubble; 7 Aug 2005; S. DeGrave leg.; GCRL 6639 • 1 ♀; Bocas del Toro, Pandora; 9.327769°N, 82.222207°W; depth 10 m, among coral rubble, 10 Aug 2021; K.N. White leg.; USNM 1703518.

###### Diagnosis.

Eyes oval. Gnathopod 1 carpus subequal to propodus. Gnathopod 2 propodus palm oblique without U-shaped excavation. Pereopods with simple dactyls; pereopod 7 basis slimmer than long, without posterodistal lobe. Uropod 3 rami three times length of peduncle, distally truncated, outer ramus with minute second article. Telson cleft, with one long and one short apical spine.

###### Distribution.

U.S.A.: North Carolina (Bynum and Fox 1977), Apalachee Bay to South Florida (LeCroy 2000), West Florida (Krapp-Schickel and Vader 2009); Pacific California (Barnard 1969b); Panama: Bocas del Toro (present study).

###### Ecology and remarks.

These amphipods are associated with sand and coral rubble at depths of 0–12 m. Panamanian specimens agree closely with the description provided by Barnard (1969b), particularly in the ornamentation and spination of the male gnathopod 2 propodus. The spines on the telson are spaced slightly differently in Panamanian specimens, but still show the same pattern.

**Figure 10.**
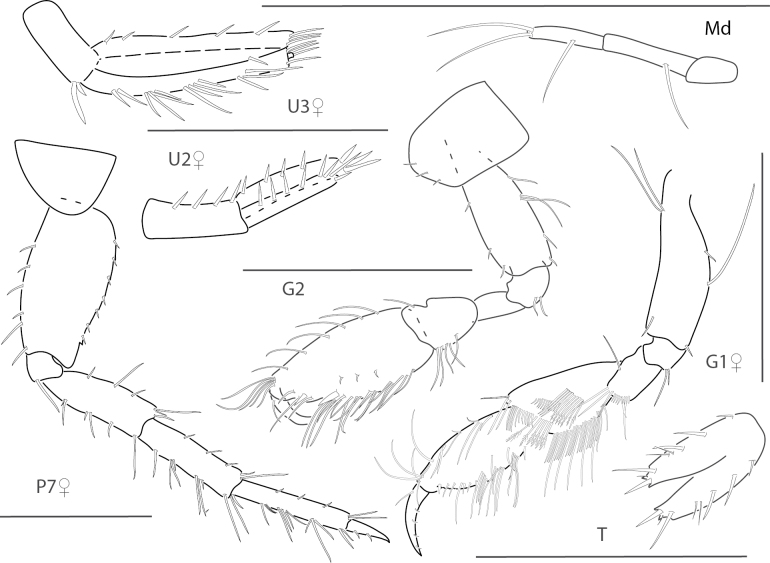
*Meximaeradiffidentia*, male, 2.5 mm, telson; female, 3.9 mm, pereopod 7, uropods 2 and 3, mandibular palp, gnathopod 1 medial, gnathopod 2 propodus with setae removed. Scale bars: 0.5 mm.

##### ﻿Genus Quadrimaera

Taxon classificationAnimaliaAmphipodaMaeridae

Krapp-Schickel & Ruffo, 2000

00B6EFBE-A36B-5D15-BC0F-A398205D22E9

###### Diagnosis.

Mandibular palp article 1 not lengthened or tooth-like; article 3 narrow. Gnathopod 1 carpus with dorso-distal excavation. Gnathopod 2 propodus palmar corner at a right angle; dactylus outer margin bare or with one seta. Pereopod dactyli bifid; pereopod 7 basis with posterodistal lobe.

##### ﻿ Quadrimaera ceres

Taxon classificationAnimaliaAmphipodaMaeridae

(Ruffo, Krapp & Gable, 2000)

1D382C55-7D3F-5698-9F9E-1FCFCD1D6E8B

Figs 11 , 28D

Maera ceres Ruffo, Krapp & Gable, 2000: 11–13, figs 4–6.

###### Material examined.

Panama • 2–3 mm • 3 ♂, 5 ♀; Bocas del Toro, Drago; 9.418056 N, 82.3375°W; depth 3 m, among coral rubble; 9 Aug 2021; K.N. White leg.; USNM 1703519.

###### Diagnosis.

Antenna 1 accessory flagellum 7-articulate. Gnathopod 1 coxa anteroventrally rounded; carpus with dorsal depression and two short and four long facial setal rows. Gnathopod 2 propodus palmar margin with shallow U-shaped excavation between two subquadrate projections, palm defined by large projection; dactylus medially expanded, smooth. Telson inner corner acutely produced, each lobe with one medium and three long apical spines.

###### Distribution.

Bermuda: St. George’s Parish (Ruffo et al. 2000); Panama: Bocas del Toro (present study).

###### Ecology and remarks.

These amphipods are associated with algae and coral rubble at depths of 0–3 m. Panamanian specimens closely resemble previously described specimens and can readily identified based on the gnathopod 2 propodus palm ornamentation.

**Figure 11.**
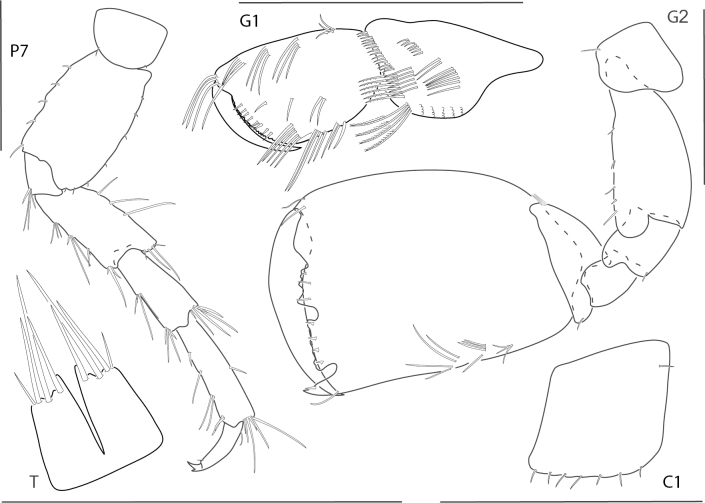
*Quadrimaeraceres*, male, 3.4 mm, pereopod 7, gnathopod 1 propodus and carpus medial, gnathopod 2 medial, telson, coxa 1. Scale bars: 0.5 mm.

##### ﻿ Quadrimaera cristianae

Taxon classificationAnimaliaAmphipodaMaeridae

Krapp-Schickel & Ruffo, 2000

2EF6A513-2E77-5302-847C-EF95756F64F0

Figs 12 , 28E

Quadrimaera cristianae Krapp-Schickel & Ruffo, 2000: 199–203, figs 3, 4.

###### Material examined.

Panama • 2–3.5 mm • 1 ♀; Bocas del Toro, Playa Bluff; 9.3905°N, 82.23725°W; depth 0 m, among algae; 5 Aug 2005; T.A. Haney leg.; GCRL 6640 • 1 ♀; Bocas del Toro, Hospital Point; 9.3336°N, 82.218883°W; depth 15 m, among coral rubble; 6 Aug 2005; S. DeGrave leg.; GCRL 6641 • 3 ♂, 5 ♀, 1 juvenile; Bocas del Toro, Hospital Point; depth 2–3 m, in sponge *Aplysiniacauliformis*; 15 June 2009; K.N. White leg.; GCRL 6642 • 1 ♂; Bocas del Toro, Hospital Point; 9.331967°N, 82.214817°W; depth 1–3 m, among *Halimeda*; 22 June 2023; K.N. White leg.; USNM 1703520 • 2 ♂, 2 ♀; Bocas del Toro, Crawl Cay; 9.245967°N, 82.136867°W; depth 1–4 m, among *Halimeda* and coral rubble; 25 June 2023; K.N. White leg.; USNM 1703521.

###### Diagnosis.

Antenna 1 accessory flagellum 6-articulate. Gnathopod 1 coxa anteroventrally rounded; carpus with slight dorsal depression and two short and four long facial setal rows. Gnathopod 2 propodus palmar margin with three U-shaped excavations increasing in size distally, two subtriangular projections, and one subrectangular projection, palm defined by large projection; dactylus medially expanded. Telson lobes inner corner acutely produced, each with four apical spines and one medio-distal plumose seta.

###### Distribution.

Turks and Caicos, Fort George Cay; Netherlands Antilles: Curaçao; Lesser Antilles: Bonaire and St. Martin; Venezuela: Margarita Island; Cayman Islands: Grand Cayman Island; Mexico: Yucatán; Brazil: Ceará State; Pernambuco State, Bahia State; Rio Grande do Norte State (Krapp-Schickel and Ruffo 2000); Panama: Bocas del Toro (present study).

###### Ecology and remarks.

These amphipods are associated with algae and coral rubble at depths of 0–15 m. Panamanian specimens closely resemble previously described specimens, with the main difference being fewer plumose setae on the telson. This species can be readily identified by the gnathopod 2 propodus palm ornamentation.

**Figure 12.**
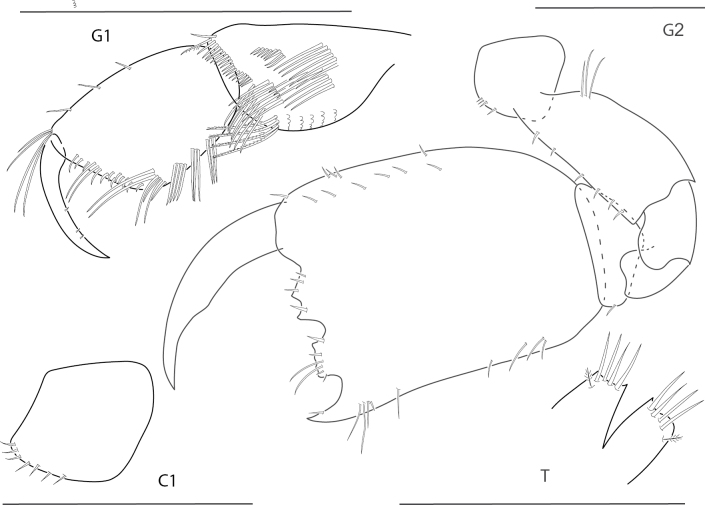
*Quadrimaeracristianae*, male, 4.9 mm, gnathopod 1 propodus, carpus, dactylus medial, telson, gnathopod 2 lateral, coxa 1. Scale bars: 0.5 mm.

##### ﻿ Quadrimaera miranda

Taxon classificationAnimaliaAmphipodaMaeridae

(Ruffo, Krapp-Schickel & Gable, 2000)

E676314D-9CE6-5F4F-885B-0EA46CA0ABE0

Figs 13 , 28F

Maera miranda Ruffo, Krapp & Gable, 2000: 15–19, figs 7, 8; LeCroy 2000: 101, fig. 148.
Quadrimaera miranda : Krapp-Schickel and Ruffo 2000: 195–196.
Maera quadrimana (non Dana 1853): Ledoyer 1986) 190–191, fig. 11.

###### Material examined.

Panama • 4–7 mm • 1 ♂, 3 ♀; Bocas del Toro, Swan Cay; 9.453333°N, 82.298333°W; depth 2–3 m; among coral rubble; 4 Aug 2005; S. DeGrave leg.; GCRL 6643 • 1 ♂, 1 ♀; Bocas del Toro, Hospital Bight; 9.304483°N, 82.172317°W; depth 0.5 m, among coral rubble; 7 Aug 2005; T.A. Haney leg.; GCRL 6644 • 2 ♀; Bocas del Toro, 100 m west of STRI dock; depth 14 m, light trap; 8 Aug 2005; T.A. Haney leg.; GCRL 6645 •1 ♀; Bocas del Toro, STRI mangroves; 9.353333°N, 82.2578°W; depth 1 m, among *Ecteinascidiaturbinata* ascidians, 11 Aug 2021; K.N. White leg.; USNM 1703522 •1 ♂; Bocas del Toro, Crawl Cay; 9.245967°N, 82.136867°W; depth 1–4 m, among *Halimeda*, 25 June 2023; K.N. White leg.; USNM 1703523 • 3 ♀; Bocas del Toro, Hospital Point; 9.333383°N, 82.218467°W; depth 0 m, buoy scraping, 26 June 2023; K.N. White leg.; USNM 1703524 • 1 ♂, 3 ♀; Bocas del Toro, STRI dock; 9.351183°N, 82.257033°W; depth 0–1 m, dock scraping, 27 June 2023; K.N. White leg.; USNM 1703525.

###### Diagnosis.

Antenna 1 accessory flagellum 6-articulate. Gnathopod 1 coxa anteroventrally rounded; carpus with minute dorsal depression and two short and three long facial setal rows. Gnathopod 2 propodus palmar margin with subrectangular projection followed by a small U-shaped excavation and one short, truncate process, palm defined by small projection; dactylus medially expanded with median point. Telson lobes apically truncate, each with five long apical spines.

###### Distribution.

Bermuda: St. George’s Parish, Sandy’s Parish (Ruffo et al. 2000); U.S.A.: Pigeon Key, FL; Turks and Caicos, Twin Cay; Netherlands Antilles: Bonaire; Lesser Antilles: St. Martin; Venezuela: Tobago Island, Los Roques Island; Brazil: Rio de Janeiro, Ilha de Fortaleza; Mexico: Yucatán (Krapp-Schickel and Ruffo 2000), Laguna de Terminos (Ledoyer 1986); Panama: Bocas del Toro (present study).

###### Ecology and remarks.

These amphipods are associated with algae, sponges, ascidians, and coral rubble at depths of 0–15 m. Panamanian specimens closely resemble previously described specimens of this species and can be readily distinguished from other species based on the gnathopod 2 palm ornamentation and broadened dactylus.

**Figure 13.**
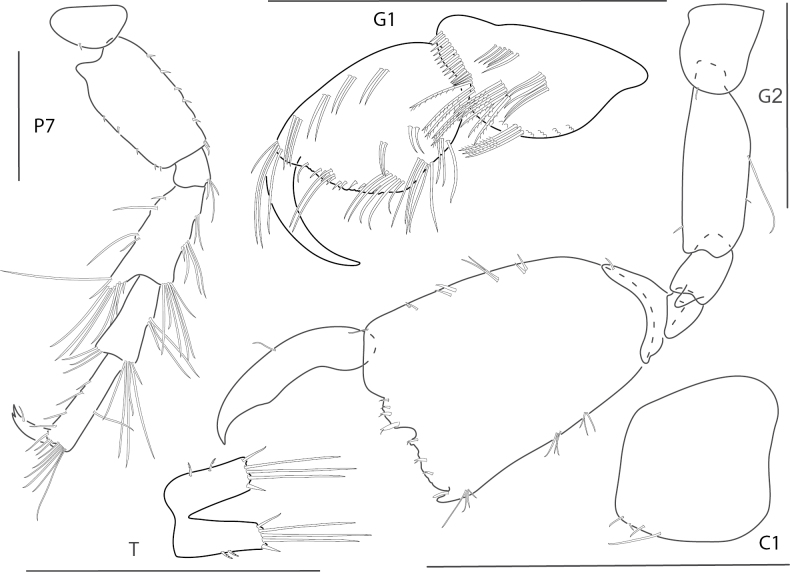
*Quadrimaeramiranda*, male, 3.3 mm, pereopod 7, gnathopod 1 propodus, carpus, dactylus medial, telson; male, 2.8 mm, gnathopod 2 lateral, coxa 1. Scale bars: 0.5 mm.

##### ﻿ Quadrimaera quadrimana

Taxon classificationAnimaliaAmphipodaMaeridae

(Dana, 1852)

E6A401E4-2546-5DFC-9076-0FA39091B0F5

Figs 14 , 28G

Gammarus quadrimanus Dana, 1852: 955–956, pl. 65, fig. 9.
Maera quadrimana : Schellenberg 1938: 45–48, figs 21, 22; LeCroy 2000: 101, fig. 147; Ruffo et al. 2000: 6–11, figs 1, 2.

###### Material examined.

Panama • 2–3.5 mm • 7 ♂, 2 ♀, 10 juvenile; Bocas del Toro, Drago; 9.418056°N, 82.3375°W; depth 2–4 m, among coral rubble; 9 Aug 2021; K.N. White leg.; USNM 1703526 • 1 juvenile; Bocas del Toro, Swan Cay; 9.4536°N, 82.300033°W; depth 1–4 m, among coral rubble; 24 Jun 2023; K.N. White leg.; USNM 1703527 • 1 ♀; Bocas del Toro, Crawl Cay; 9.245967°N, 82.136867°W; depth 1–4 m, among *Halimeda*; 25 June 2023; K.N. White leg.; USNM 1703528.

###### Diagnosis.

Antenna 1 accessory flagellum 6-articulate. Gnathopod 1 coxa anteroventrally rounded; carpus with distinct dorsal depression and two short and three long facial setal rows. Gnathopod 2 propodus palmar margin with three U-shaped excavations, increasing in size distally, two small subtriangular projections, and one large truncate projection, palm defined by large projection; dactylus smooth, not expanded. Telson lobes apically truncate, inner margins acute, each with four or five medium-to-long apical spines.

###### Distribution.

Fiji Islands (Dana 1853); Red Sea (Ruffo 1969); Madagascar (Ledoyer 1972, 1982); Great

Barrier Reef, Australia (Berents 1983); Gilbert Islands (Schellenberg 1938); Micronesia (J.L. Barnard 1965); Hawaiian Islands (J.L. Barnard 1970, 1971); Bermuda: St. George’s Parish, Devonshire Parish (Ruffo et al. 2000); U.S.A.: Florida (Nelson 1995; Thomas 1993; LeCroy 2000); Mexico: Yucatán (McKinney 1977); Brazil (Wakabara et al. 1991; Wakabara and Serejo 1998); Panama: Bocas del Toro (present study).

**Figure 14.**
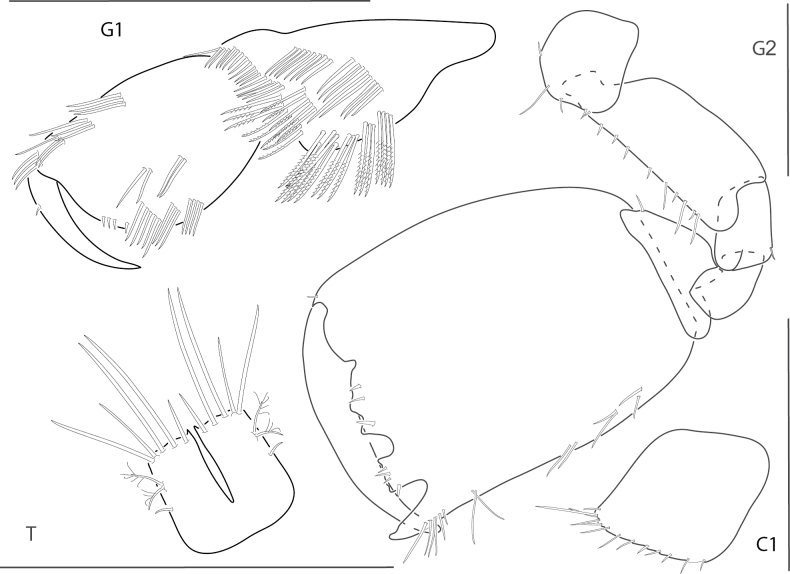
*Quadrimaeraquadrimana*, male, 3.8 mm, gnathopod 1 propodus, carpus, dactylus medial, gnathopod 2 lateral, coxa 1, telson. Scale bars: 0.5 mm.

###### Ecology and remarks.

These amphipods are associated with algae and coral rubble at depths of 0–10 m. Panamanian specimens closely resemble the description of specimens from Bermuda, with the gnathopod 2 propodus palmar margin showing a slightly more truncate projection than illustrated by Ruffo et al. (2000).

##### ﻿ Quadrimaera sarae

Taxon classificationAnimaliaAmphipodaMaeridae

Krapp-Schickel & Ruffo, 2000

37F008EB-BFD9-5752-94A2-7E35B72659A7

Figs 15 , 29A

Quadrimaera sarae Krapp-Schickel & Ruffo, 2000: 206–213, figs 8–10.

###### Material examined.

Panama • 4–5 mm • 2 ♂, 2 ♀; Bocas del Toro, Swan Cay; 9.453333°N, 82.298333°W; depth 2–3 m; among coral rubble; 4 Aug 2005; S. DeGrave leg.; GCRL 6646 • 1 ♂; Bocas del Toro, Drago; 9.418056 N, 82.3375°W; depth 3 m, among coral rubble; 9 Aug 2021; K.N. White leg.; USNM 1703529.

###### Diagnosis.

Antenna 1 accessory flagellum 7-articulate. Gnathopod 1 coxa anteroventrally produced; carpus elongate with slight dorsal depression and two short and three long facial setal rows. Gnathopod 2 propodus palmar margin with U-shaped excavation surrounded by one subquadrate and one quadrate projection, palm defined by small projection; dactylus medially expanded. Pereopods 3 and 4 dactyli simple; pereopods 5–7 dactyli bifid. Telson, lobes apically excavated, each with four long apical spines.

###### Distribution.

Turks and Caicos, Fort George Cay; Mexico: Yucatán; Venezuela: Tobago Island (Krapp-Schickel and Ruffo 2000); Panama: Bocas del Toro (present study).

###### Ecology and remarks.

These amphipods are associated with coral rubble at depths of 0.3–3 m. Panamanian specimens closely resemble previously described specimens, including the characteristic gnathopod 2 propodus palm, simple pereopods 3 and 4 dactyli, and bifid pereopods 5–7 dactyli, which are unique to this species. The excavation on the gnathopod 2 propodus is larger in our 4.2 mm male than shown in the holotype (4.7 mm male) and there are more than three spines on the pereopods 3 and 4 bases in Panamanian specimens, but given the striking similarity in every other character, we are considering this as a regional variation.

**Figure 15.**
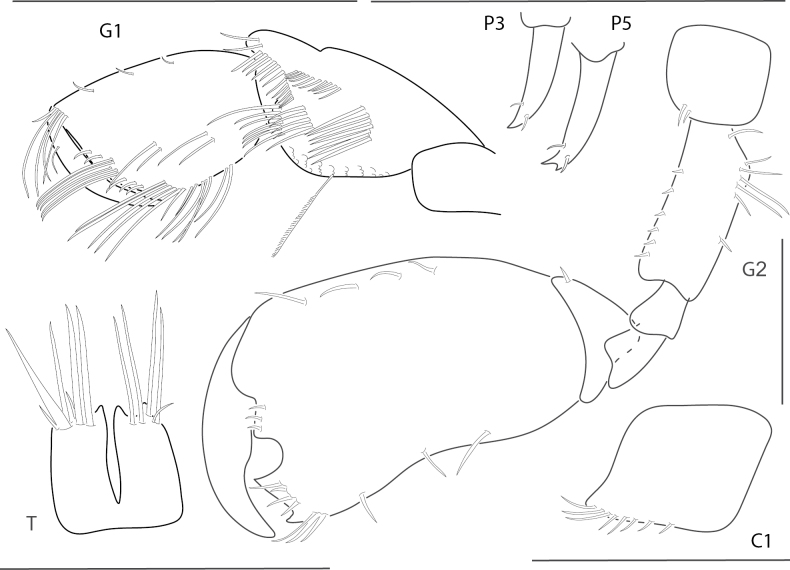
*Quadrimaerasarae*, male, 4.2 mm, gnathopod 1 propodus, carpus, dactylus medial, pereopod 3 dactylus, pereopod 5 dactylus, gnathopod 2 medial, coxa 1, telson. Scale bars: 0.5 mm.

##### ﻿ Quadrimaera yemanjae

Taxon classificationAnimaliaAmphipodaMaeridae

Alves, Neves & Johnson, 2018

4130F7DB-75E4-51DB-8147-C36D019523C0

Figs 16 , 29B

Quadrimaera yemanjae Alves, Neves & Johnson, 2018: 569–575, figs 2–7.

###### Material examined.

Panama • 2–4.5 mm • 10 ♂, 4 ♀; Bocas del Toro, Swan Cay; 9.453333°N, 82.298333°W; depth 2–3 m, among coral rubble; 4 Aug 2005; S. DeGrave leg.; GCRL 6647 • 1 ♀, 9 juvenile; Bocas del Toro, TAH 001, Aug 2005; T.A. Haney leg.; GCRL 6648 • 1 ♂, Bocas del Toro, Drago; 9.418056°N, 82.3375°W; depth 2–4 m, among coral rubble; 9 Aug 2021; K.N. White leg.; USNM 1703530 • 2 ♀; Bocas del Toro, Crawl Cay; 9.245967°N, 82.136867°W; depth 1–4 m, among coral rubble; 25 June 2023; K.N. White leg.; USNM 1703531.

###### Diagnosis.

Antenna 1 accessory flagellum 6-articulate, distal article minute. Gnathopod 1 coxa anteroventrally rounded; carpus with deep dorsal depression and two short and three long facial setal rows. Gnathopod 2 propodus palmar margin with two small U-shaped excavations separated by a subacute process, followed distally by a large truncate process and one large U-shaped excavation, palm defined by large projection; dactylus medially smooth, slightly expanded. Telson lobes apically truncate, inner margins acute, each with four long apical spines.

###### Distribution.

Brazil: Rio Grande do Norte State (Alves et al. 2018); Panama: Bocas del Toro (present study).

###### Ecology and remarks.

These amphipods are associated with *Halimeda* and coral rubble at depths of 0–4 m. Panamanian specimens closely resemble specimens described from Brazil (4.5 mm), with slightly less acute projections on the palm of gnathopod 2 propodus. This can most likely be attributed to the smaller size of the Panamanian specimens (3.8 mm).

**Figure 16.**
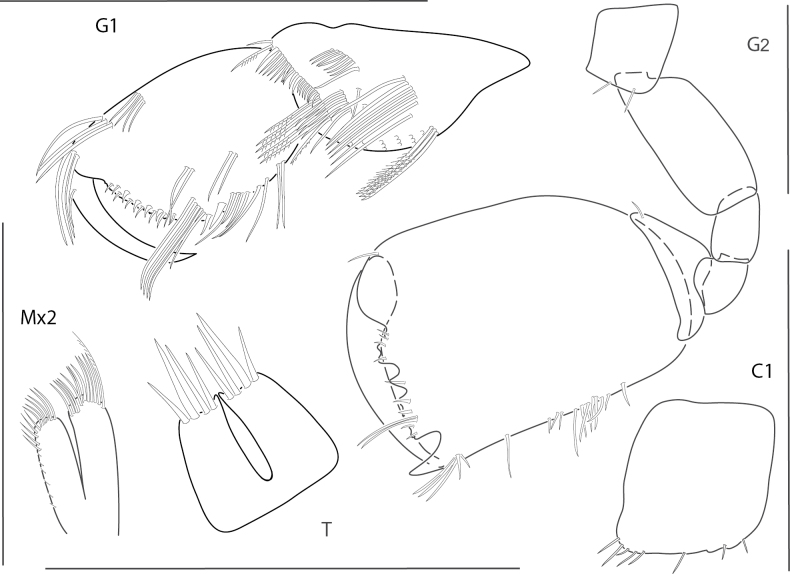
*Quadrimaerayemanjae*, male, 3.8 mm, gnathopod 1 propodus, carpus, dactylus medial, gnathopod 2 lateral, coxa 1, maxilla 2, telson. Scale bars: 0.5 mm.

#### ﻿Family Melitidae Bousfield, 1973

##### ﻿Genus Dulichiella

Taxon classificationAnimaliaAmphipodaMelitidae

Stout, 1912

78B28FDA-0F85-5B8B-ADC4-A1E3CA1F1AA9

###### Diagnosis.

Male gnathopod 2 large, asymmetrical, propodus with distolateral crown of spines; female gnathopod 2 equal in size. Pereopods 5–7 dactyli each with accessory spine. Pleosome and urosome with dorsolateral spines. Uropod 3 inner ramus minute; outer ramus 2-articulate. Telson, deeply cleft, tapering to an acute point.

##### ﻿ Dulichiella anisochir

Taxon classificationAnimaliaAmphipodaMelitidae

(Krøyer, 1845)

C232C53A-3705-5D10-A7D4-5E39C91DE26F

Figs 17 , 29C

Melitaanisochir Krøyer, 1845: 317, pl. II, fig. 1a–p; Dana 1852: 968, pl. 66, fig. 8a–d.
Dulichiella anisochir : Lowry and Springthorpe 2007: 10–12, figs 3–6.

###### Material examined.

Panama • 4–6 mm • 4 ♂, 2 ♀, 4.0 mm; Bocas del Toro, Crawl Cay; 9.2475°N, 82.1290°W; depth 5 m, among coral rubble; 12 Aug 2021; K.N. White leg.; USNM 1703532.

###### Diagnosis.

Gnathopod 1 coxa anteroventral corner produced, rounded, anterior margin concave. Gnathopod 2 propodus distolateral crown with three rounded spines; dactylus apically blunt, overlapping corner of propodus. Pereopods 6 and 7 carpus and propodus without bunches of long slender setae. Epimeron 1 posteroventral corner acute; epimeron 3 posterodistal margin serrate.

###### Distribution.

Brazil: Rio de Janeiro to Lagoa dos Patos (Krøyer 1845; Lowry and Springthorpe 2007); Panama: Bocas del Toro (present study).

###### Ecology and remarks.

These amphipods are associated with soft bottoms and coral rubble at depths of 0–30 m. Panamanian specimens closely resemble previously described specimens, with the exception of a less serrate distal margin on epimeron 3. This difference can most likely be attributed to the size difference of our specimens (4.4 mm) and the lectotype (10.7 mm) described by Lowry and Springthorpe (2007).

**Figure 17.**
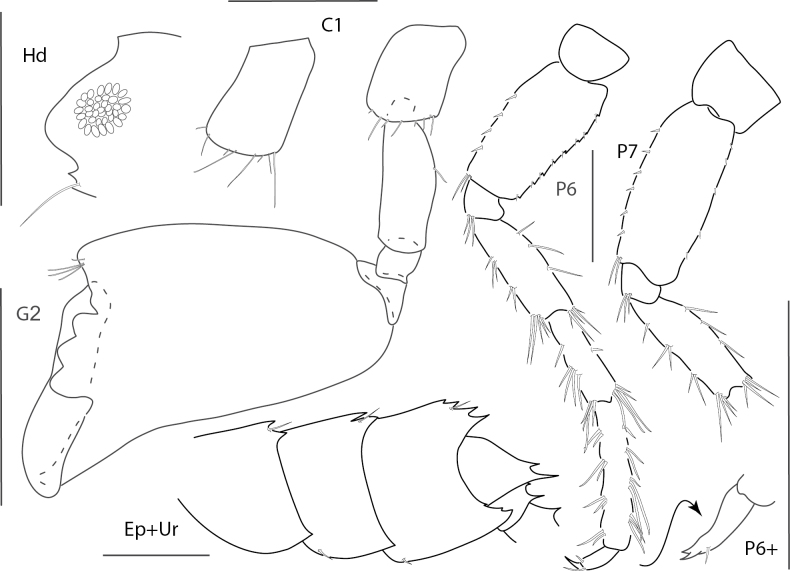
*Dulichiellaanisochir*, male, 4.4 mm, head, coxa 1, gnathopod 2 lateral, epimeron and urosome, pereopods 6 and 7, dactylus enlarged. Scale bars: 0.5 mm.

##### ﻿ Dulichiella lecroyae

Taxon classificationAnimaliaAmphipodaMelitidae

Lowry & Springthorpe, 2007

388E8C91-7F4E-50DF-B475-1E3EC3ED700B

Figs 18 , 29D

*Melitafresnelli*Kunkel 1910: 31–34, fig. 11; Pearse 1912: 371.
Dulichiella sp. A: LeCroy 2000: 78, fig. 126.
Dulichiella lecroyae Lowry & Springthorpe, 2007: 34–38, figs 25–28.

###### Material examined.

Panama • 5–10 mm • 1 ♂, 1 ♀; Bocas del Toro, Swan Cay; 9.453333°N, 82.298333°W; depth 2–3 m, in orange sponge; 4 Aug 2005; S. DeGrave leg.; GCRL 6649 • 4 ♂, 1 ♀; Bocas del Toro, Crawl Cay; 9.250467°N, 82.131617°W; depth 10 m, in sponge; 7 Aug 2005; S. DeGrave leg.; GCRL 6650 • 1 ♀; Bocas del Toro, Punta Caracol; depth 1 m, in *Lissodendoryxcolumbiensis* sponge, 9 June 2009; K. Hultgren leg.; GCRL 6651 • 5 ♂, 4 ♀; Bocas del Toro, STRI Point; 9.34872°N, 82.26258°W; depth 12 m, among coral rubble, 6 Aug 2021; K.N. White leg.; USNM 1703533• 1 ♂, 3 ♀; Bocas del Toro, Juan Point; 9.3015°N, 82.29404°W; depth 10 m, among coral rubble, 7 Aug 2021; K.N. White leg.; USNM 1703534 • 12 ♂, 5 ♀; Drago; 9.418056°N, 82.3375°W; depth 2–3 m, among coral rubble, 9 Aug 2021; K.N. White leg.; USNM 1703535 • 1 ♀; Bocas del Toro, San Cristobal; 9.2625°N, 82.235°W; depth 15 m, among coral rubble, 10 Aug 2021; K.N. White leg.; USNM 1703536 • 1 ♂; Bocas del Toro, Pandora; 9.327769°N, 82, 222207°W; depth 10 m, among coral rubble, 10 Aug 2021; K.N. White leg.; USNM 1703537 • 1 ♂; Bocas del Toro, Crawl Cay; 9.2376°N, 82.1438°W; depth 1.5–3 m, among coral rubble, 11 Aug 2021; K.N. White leg.; USNM 1703538.

###### Diagnosis.

Gnathopod 1 coxa anteroventral corner not produced, anterior margin straight. Gnathopod 2 propodus distolateral crown with four rounded or subacute spines; dactylus apically hooked, fitting into posterodistal corner of propodus. Pereopods 6 and 7 carpus and propodus without bunches of long slender setae. Epimeron 1 posteroventral corner subquadrate; epimeron 3 posterodistal margin smooth.

###### Distribution.

U.S.A.: Gulf of Mexico, South Florida, Cedar Keys, Dry Tortugas, South Carolina, Georgia (LeCroy 2000; Lowry and Springthorpe 2007); Bermuda: Flatts Village, Castle Harbor, Harrington Sound (Kunkel 1910); Panama: Bocas del Toro (present study).

###### Ecology and remarks.

These amphipods are associated with sponges and coral rubble at depths of 0–12 m. Panamanian specimens closely resemble previously described specimens, with slight variation in the anteroventral margin of the head. Panamanian specimens show a minutely bifid notch rather than a single acute point.

**Figure 18.**
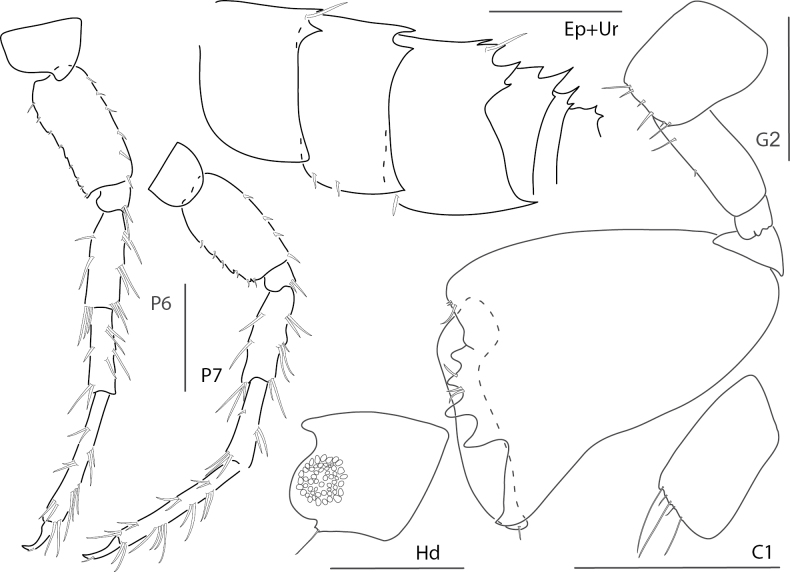
*Dulichiellalecroyae*, male, 3.6 mm, pereopods 6 and 7, epimeron and urosome, gnathopod 2 lateral, head, coxa 1. Scale bars: 0.5 mm.

##### ﻿﻿Genus Melita

Taxon classificationAnimaliaAmphipodaMelitidae

Leach, 1814

A9ACF1B7-257E-5C29-AB73-2B7F7BC7C697

###### Diagnosis.

Male gnathopod 2 large, symmetrical; female gnathopod 2 smaller than in male. Pereopods 5–7, dactyli without accessory spines. Pleosome without serrations. Uropod 3 inner ramus minute; outer ramus 1-articulate. Telson deeply cleft, sides straight or convex, tapering to a point, with apical spines.

##### ﻿Melita planaterga

Taxon classificationAnimaliaAmphipodaMelitidae

Kunkel, 1910

31D83ADF-9AEF-5B45-8B92-E13AD51E6C47

Figs 19 , 29E

*﻿Melitaplanaterga* Kunkel, 1910: 34–37, fig. 12; LeCroy 2000: 115, fig. 149.

###### Material examined.

Panama • 4–9 mm • 7 ♂, 5 ♀; Bocas del Toro, Swan Cay; 9.453333°N, 82.298333°W; depth 2–3 m, among algae; 4 Aug 2005; S. DeGrave leg.; GCRL 6652 • 1 ♀; Bocas del Toro, San Cristobal; 9.284977°N, 82.294533°W; depth 1–3 m, among *Dictyota*; 21 June 2023; K.N. White leg.; USNM 1703539 • 6 ♂, 5 ♀; Bocas del Toro, Drago; 9.413433°N, 82.33335°W; depth 1–3 m, among *Halimeda*, red algae and coral rubble; 23 June 2023; K.N. White leg.; USNM 1703540 • 1 ♂, 1 ♀ Bocas del Toro, Swan Cay; 9.4536°N, 82.300033°W; depth 2 m, among coral rubble; 24 June 2023; K.N. White leg.; USNM 1703541.

###### Diagnosis.

Male antennae without bottle-brush setae. Male gnathopod 2 propodus ovate, palm densely setose, setae shorter than propodus length; female gnathopod 2 smaller and less setose than in male. Female coxa 6 with lateral ridge at base of hook, anteroventral angle flattened or notched. Urosome segment 1 posterodorsal margin with single median process; urosome segment 2 posterior margin smooth, each side with single dorsolateral spine. Telson lobes apically subacute with long terminal spines.

###### Distribution.

U.S.A.: Gulf of Mexico, Florida Keys (Lazo-Wasem and Gable 1987; LeCroy 2000); Bermuda: Flatts Village (Kunkel 1910); Mexico: Terminos Lagoon, Bay of Campeche, Mexico (Ledoyer 1986); Panama: Bocas del Toro (present study).

###### Ecology and remarks.

These amphipods are associated with algae and coral rubble at depths of 0–3 m. Panamanian specimens closely resemble previously described specimens. Females can be identified easily by the structure of coxa 6 and males have a heavily setose gnathopod 2 propodus. The dark pigmentation is also characteristic of this species. Panamanian specimens ranged from having dark pigment bands as described by Kunkel (1910) to being almost completely dark blue (more so than in Fig. 29E).

**Figure 19.**
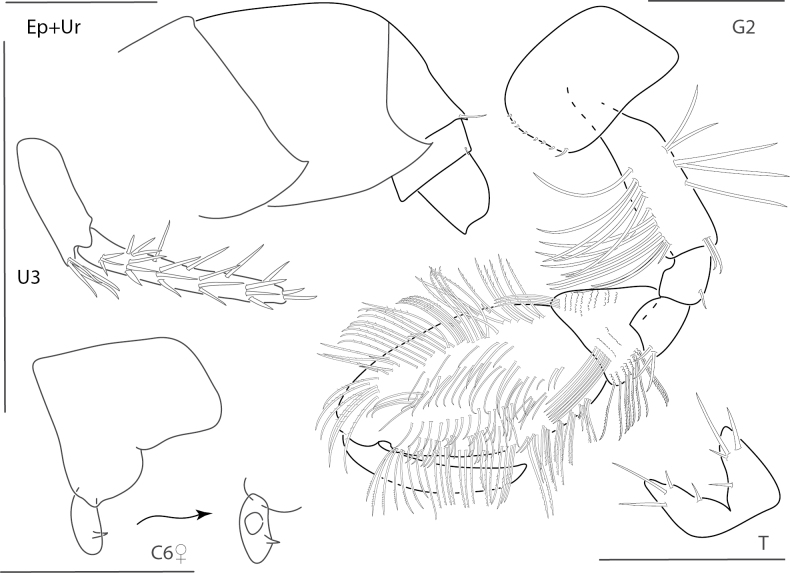
*Melitaplanaterga*, male, 5.1 mm, uropod 3, telson, gnathopod 2 medial, some setae removed from carpus, epimeron and urosome; female, 4.6 mm, coxa 6 lateral, coxa 6 medial hook. Scale bars: 0.5 mm.

### ﻿Superfamily Calliopioidea Sars, 1895

#### ﻿Family Hornelliidae d’Udekem d’Acoz, 2010

##### ﻿Genus Hornellia

Taxon classificationAnimaliaAmphipodaHornelliidae

Walker, 1904

090EB658-B8F8-5EE7-A876-E602117D6D6E

###### Diagnosis.

Head, without rostrum. Antenna 1 accessory flagellum present. Gnathopods 1 and 2 subequal and similar. Pereopods 5–7 long and slender. Pleosome and urosome with postero-dorsal teeth. Uropod 3 biramous, rami subequal in length. Telson long and deeply cleft.

##### ﻿ Hornellia tequestae

Taxon classificationAnimaliaAmphipodaHornelliidae

Thomas & J.L. Barnard, 1986

8E2B67B1-EC8E-5AA7-8EF8-7BE55BBA6D59

Figs 20 , 29F

Hornellia (Metaceradocus) tequestae Thomas & J.L. Barnard, 1986a: 478–483, figs 1–3; LeCroy 2007: 591.

###### Material examined.

Panama • 2–3 mm 1 ♂; Bocas del Toro, Crawl Cay; 9.237675°N, 82.143833°W; depth 2–3 m, among *Halimeda*; 11 Aug 2021; K.N. White leg.; USNM 1703542 • 1 ♂, 2 ♀, Bocas del Toro, Hospital Point; 9.331967°N, 82.214817°W; depth 1–3 m, coral rubble; 22 June 2023; K.N. White leg.; USNM 1703543 • 2 ♂, 2 ♀; Bocas del Toro, Crawl Cay; 9.245967°N, 82.136867°W; depth 1–4 m, coral rubble; 25 June 2023; K.N. White leg.; USNM 1703544 • 2 ♂, 4 ♀; Bocas del Toro, Crawl Cay; 9.250217°N, 82.131767°W; depth 5–13 m, coral rubble; 29 June 2023; K.N. White leg.; USNM 1703545.

###### Diagnosis.

Antenna 1 accessory flagellum 4-articulate. Gnathopod 1 carpus posterior margin densely setose. Gnathopod 2 not sexually dimorphic, propodus elongate, palm oblique, smooth. Pereopods 5–7 bases posterior margins strongly serrate; pereopod 7 basis without posterodistal lobe. Epimera 1–3 posterior margins smooth, each with well-developed posteroventral tooth. Telson 1.4 × longer than broad, nearly cleft to base, lobes apically acute with two setae.

###### Distribution.

U.S.A.: Southeastern Gulf of Mexico (LeCroy 2007), Florida Keys (Thomas and Barnard 1986a); Panama: Bocas del Toro (present study).

###### Ecology and remarks.

These amphipods are associated with algae and coral rubble at depths of 0–45 m. Panamanian specimens closely resemble previously described specimens and are readily identified by the distinctly serrate posterodorsal margins of the pleosome and urosome, large posteroventral tooth on each epimeron, and the shape of the telson.

**Figure 20.**
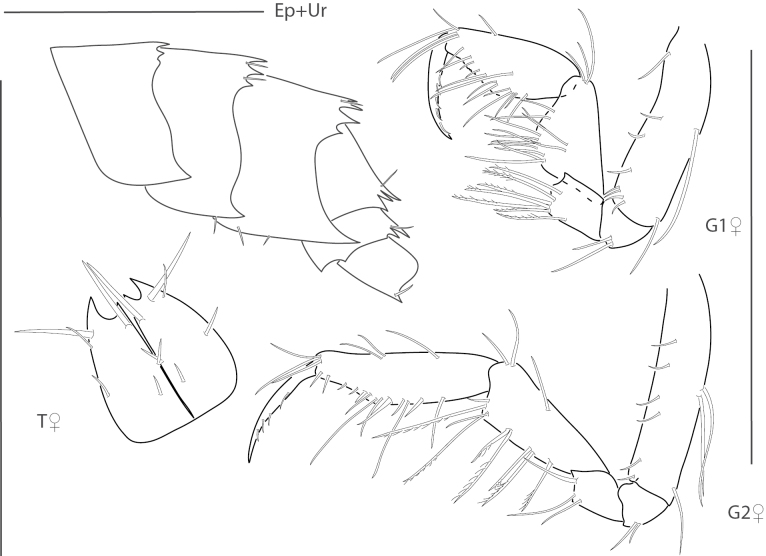
*Hornelliatequestae*, male, 1.8 mm, epimeron and urosome; female, 2.2 mm, telson, gnathopods 1 and 2 medial. Scale bars: 0.5 mm.

#### ﻿Family Megaluropidae Thomas & J.L. Barnard, 1986

##### ﻿Genus Gibberosus

Taxon classificationAnimaliaAmphipodaMegaluropidae

Thomas & J.L. Barnard, 1986

C2E1EA37-309C-5650-859D-B82E0EB16BDB

###### Diagnosis.

Head rostrum short; ocular lobe with acute cusp. Antenna 1 accessory flagellum 2-articulate. Gnathopod 2 merus with large distal lobe. Uropod 1 peduncle with interramal tooth. Telson with long spines.

##### ﻿ Gibberosus devaneyi

Taxon classificationAnimaliaAmphipodaMegaluropidae

Thomas & J.L. Barnard, 1986

F3F1D40A-19A9-5AA4-8571-0B71346179BE

Figs 21 , 30A

Gibberosus devaneyi Thomas & J.L. Barnard, 1986b: 469–475, figs 11, 13–15.

###### Material examined.

Panama • 2–3 mm • 6 ♂, 56 ♀; Bocas del Toro, Cayo Solarte;; 9.3336°N, 82.218883°W; depth 0.1–1 m, in sand; 7 Aug 2005; S.E. LeCroy leg.; GCRL 6653 • 1 ♂; Bocas del Toro, Drago; 9.413433°N, 82.33335°W; depth 1–3 m, among *Halimeda*, red algae and coral rubble; 23 June 2023; K.N. White leg.; USNM 1703546 • 1 ♂, 5 ♀; Bocas del Toro, Crawl Cay; 9.250217°N, 82.131767°W; depth 5–13 m, coral rubble; 29 June 2023; K.N. White leg.; USNM 1703547.

###### Diagnosis.

Head ocular lobe with subacute cusp. Pleosome segment 3 and urosome segment 2 with dorsal serrations, other segments smooth, lacking dorsal spines. Epimeron 3 smooth. Uropod 3 rami continually lined with spines. Telson lobes with several dorsal and two apical spines.

###### Distribution.

U.S.A.: La Jolla, California; Peru: Chincha Island (Thomas and Barnard 1986b); Panama: Bocas del Toro (present study).

###### Ecology and remarks.

These amphipods are found in sand at depths of 0–18 m. Panamanian specimens closely resemble specimens described from the eastern Pacific with slight variation in the following characters: subacute anterior head margin (acute in Pacific material) and slightly more serrate posterodorsal margin on pleon segment 3. All other characters match well, specifically the smooth posterior margin of epimeron 3 and the continually spinose margins of uropod 3 rami.

**Figure 21.**
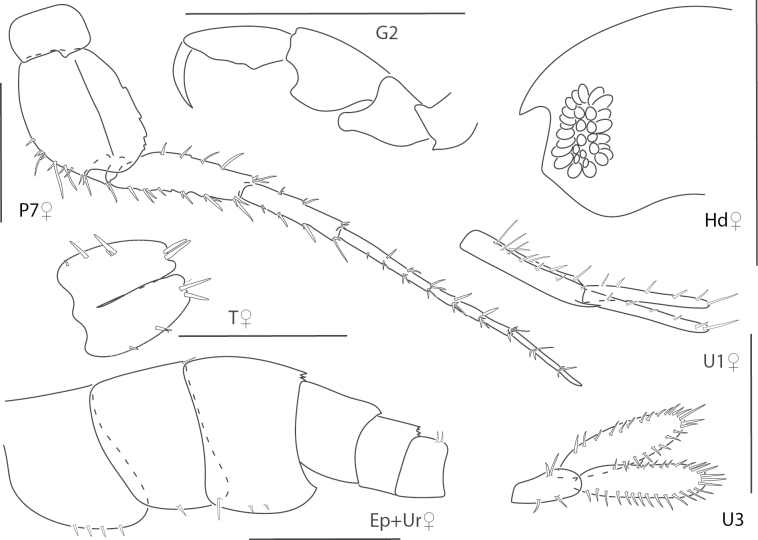
*Gibberosusdevaneyi*, female, 3.7 mm, pereopod 7, telson, epimeron and urosome, head, uropod 1; male, 3.0 mm, gnathopod 2, setae removed, uropod 3. Scale bars: 0.5 mm.

##### ﻿ Gibberosus myersi

Taxon classificationAnimaliaAmphipodaMegaluropidae

(McKinney, 1980)

29F98E1B-F237-5C7A-AC2B-D7EC774B5C25

Figs 22 , 30B

Megaluropus longimerus : Barnard 1962: 103, figs 17o–q (non Megaluropuslongimerus Schellenberg, 1925).
Megaluropus sp.: Camp et al. 1977: 17–18.
Megaluropus myersi McKinney, 1980: 93–98, figs 5–7.
Gibberosus myersi : Thomas and Barnard 1986b: 464–469, figs 6, 12; LeCroy 2007: 590.
Gibberosus sp. A: Rakocinski et al. 1993: 102.
Gibberosus cf. myersi : Rakocinski et al. 1996: 350.

###### Material examined.

Panama • 2–5 mm • 2 ♀; Bocas del Toro, Crawl Cay; 9.237675°N, 82.143833°W; depth 2–3 m, in sand; 11 Aug 2021; K.N. White leg.; USNM 1703548 • 1 ♂, 3 ♀, Bocas del Toro, Drago; 9.413433°N, 82.33335°W; depth 1–3 m, in sand; 23 June 2023; K.N. White leg.; USNM 1703549 • 1 ♂, 8 ♀; Bocas del Toro, Drago; 9.417183°N, 82.324783°W; depth 0–1 m in sand; 27 June 2023; K.N. White leg.; USNM 1703550.

###### Diagnosis.

Head ocular lobe with acute cusp. Pleosome segment 3 and urosome segments 1 and 2, with dorsal serrations; urosome segment 2, with one or two dorsal spines. Epimeron 3 serrate. Uropod 3 peduncle with facial spines; rami with sparse marginal spines. Telson each lobe with one dorsal and two apical spines.

###### Distribution.

U.S.A.: South Carolina to the Florida Keys; southwestern Gulf of Mexico, Tampa Bay, Perdido Key, British Columbia to La Jolla, California (Thomas and Barnard 1986b; Rakocinski et al. 1993, 1996; LeCroy 2007); Peru: Afuera; Costa Rica: Puerto Culebra; Brazil: llha Sao Sebastiao (Thomas and Barnard 1986b); Panama: Culebra Island (Thomas and Barnard 1986b), Bocas del Toro (present study).

###### Ecology and remarks.

These amphipods are found in sand at depths of 0–29 m. Panamanian specimens closely resemble previously described specimens. See Thomas and Barnard (1986b) for a discussion of the variation in this species. Caribbean Panamanian specimens most closely resemble specimens from Brazil, Peru, Costa Rica, and Queen Charlotte, and Coronados based on having a smooth dorsal margin on pleonites 2 and 5, a thin posterior most facial spine on peduncle of uropod 1, and epimeron 2 without facial spines.

**Figure 22.**
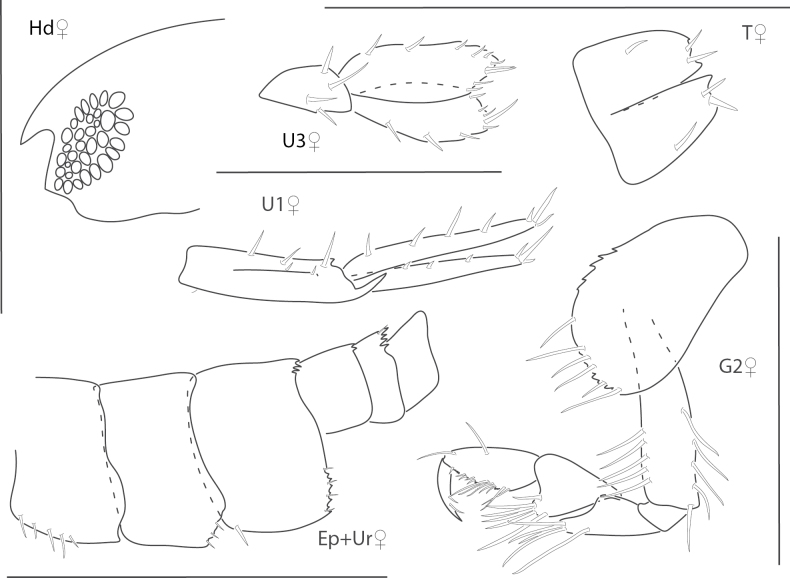
*Gibberosusmyersi*, female, 2.1 mm, head, uropods 1 and 3, telson, epimeron and urosome, gnathopod 2 lateral. Scale bars: 0.5 mm.

##### ﻿Genus Resupinus

Taxon classificationAnimaliaAmphipodaMegaluropidae

Thomas & J.L. Barnard, 1986

09E21816-AC5D-5EB6-B120-39A015B4A206

###### Diagnosis.

Head rostrum long; ocular lobe rounded. Antenna 1 accessory flagellum 1-articulate. Gnathopod 2 merus without distal lobe. Uropod 1 peduncle without interramal tooth. Telson with small spines (if present).

##### ﻿ Resupinus spinicaudatus

Taxon classificationAnimaliaAmphipodaMegaluropidae

Thomas & J.L. Barnard, 1986

293447F9-FAC4-5F25-A45A-A2978FD6C882

Figs 23 , 30C

Resupinus spinicaudatus Thomas & J.L. Barnard, 1986b: 445–454, figs 1–5.

###### Material examined.

Panama • 2–2.5 mm • 2 ♂, 6 ♀; Bocas del Toro, Drago; 9.413433°N, 82.33335°W; depth 0–1 m, in sand; 23 June 2023; K.N. White leg.; USNM 1703551.

###### Diagnosis.

Head eye not filling entire ocular lobe. Pleosome segments 2 and 3 with dorsal serrations. Urosome segments dorsally smooth. Epimera 1–3 with sparse facial setae; epimeron 3 posterior margin with sparse, shallow serrations. Telson covered with dorsal prickle spines.

###### Distribution.

Belize: Sitee Point (Thomas and Barnard 1986b); Panama: Bocas del Toro (present study).

###### Ecology and remarks.

These amphipods are found in sand at depths of 0.75–1.2 m. Panamanian specimens closely resemble previously described specimens and can be easily identified based on having smooth pleonites 4 and 5, sparsely serrate epimeron 3, and dorsally spinose telson.

**Figure 23.**
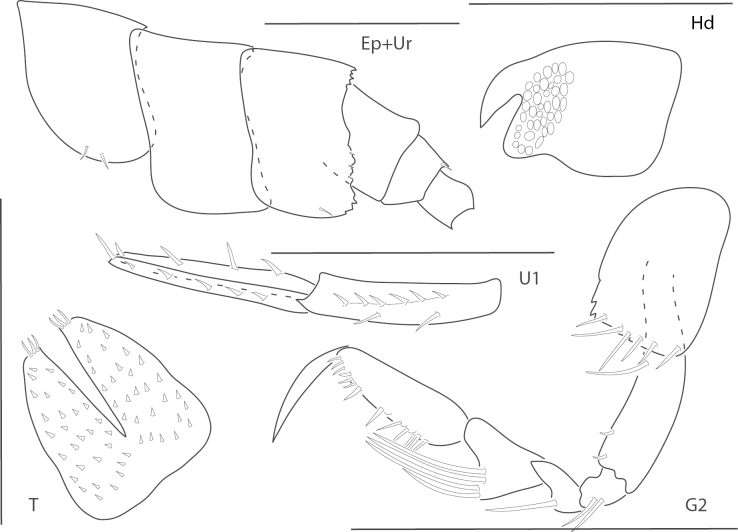
*Resupinusspinicaudatus*, male, 2.4 mm, epimeron and urosome, head, uropod 1, telson, gnathopod 2 lateral. Scale bars: 0.5 mm.

#### ﻿Family Pontogeneiidae Stebbing, 1906

##### ﻿Genus Eusiroides

Taxon classificationAnimaliaAmphipodaPontogeneiidae

Stebbing, 1888

FBB030E2-496E-54DC-8B49-CFED5B5C04A0

###### Diagnosis.

Antenna 1 accessory flagellum 1-articulate. Rostrum short. Gnathopods 1 and 2 propodus palmar margins lined with stout peg-like spines. Epimeron 3 posterior margin serrate. Uropod 2 rami subequal with length of uropods 1 and 3.

##### ﻿ Eusiroides yucatanensis

Taxon classificationAnimaliaAmphipodaPontogeneiidae

McKinney, 1980

E00F5063-9009-58B2-908D-661238C044BE

Figs 24 , 30D

Eusiroides yucatanensis McKinney, 1980: 89–93, figs 3, 4; Diaz and Martin 2000: 767.

###### Material examined.

Panama • 5–7 mm • 1 ♂, 1 ♀, 2 juvenile; Bocas del Toro, Crawl Cay; 9.2376°N, 82.1438°W; depth 1.5–3 m, among coral rubble, 11 Aug 2021; K.N. White leg.; USNM 1703552.

###### Diagnosis.

Pereopods 5–7 basis crenulate; propodus with spine formula 2, 2, 2, 2, and two locking spines. Epimera 1 and 2 smooth, posteroventral corner with acute point; epimeron 3 posterior margin with three serrations. Uropod 3 peduncle 1/2 as long as rami. Telson subtriangular, longer than wide, apices of lobes subacute.

###### Distribution.

Mexico: Yucatan (McKinney 1980); Venezuela: Puerto Viejo (Diaz and Martin 2000); Panama: Bocas del Toro (present study).

###### Ecology and remarks.

These amphipods are associated with algae and coral rubble at depths of 0–3 m. Panamanian specimens closely resemble previously described specimens and can be easily identified based on the smooth epimera 1 and 2, epimeron 3 having three distinct serrations, and the length of uropod 3 peduncle.

**Figure 24.**
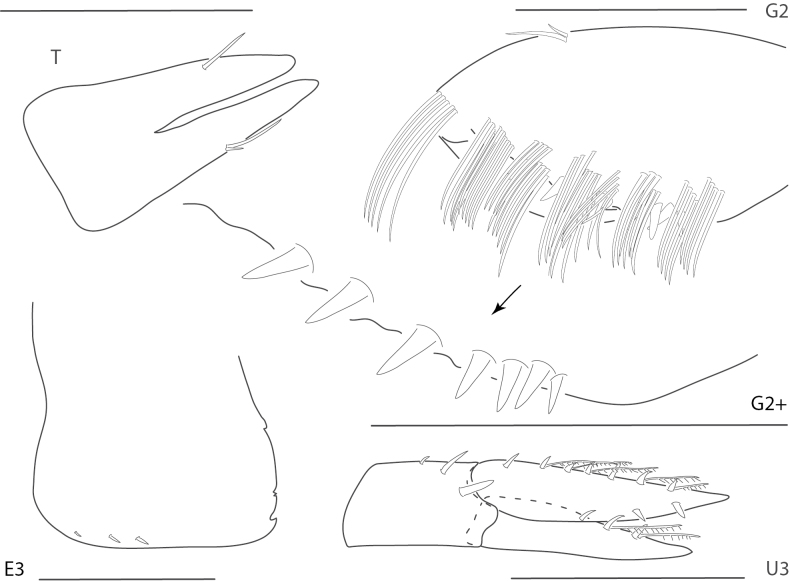
*Eusiroidesyucatanensis*, male, 6.9 mm, telson, gnathopod 2 propodus medial, gnathopod 2 propodus medial enlarged, setae removed, epimeron 3, uropod 3. Scale bars: 0.5 mm.

##### ﻿Genus Nasageneia

Taxon classificationAnimaliaAmphipodaPontogeneiidae

Barnard & Karaman, 1987

80BF888C-7496-5F2D-A5BB-CB725E08C994

###### Diagnosis.

Antenna 1 without accessory flagellum. Rostrum reaching ~ 1/2 length of first article of antenna 1 peduncle. Gnathopods 1 and 2 propodus relatively small, palmar margin lined with slender spines. Epimeron 3 posterior margin serrate. Telson subrectangular, slightly longer than wide, apices of lobes rounded or subtruncate.

##### ﻿ Nasageneia bacescui

Taxon classificationAnimaliaAmphipodaPontogeneiidae

Ortiz & Lalana, 1994

B6151886-8E0D-5029-995F-30BB9999DEB7

Figs 25 , 30E

Nasageneia bacescui Ortiz & Lalana, 1994: 285–291, figs 1–5; LeCroy 2007: 512, fig. 451.

###### Material examined.

Panama • 2.5–5 mm • 1 ♂ Bocas del Toro, Drago; 9.413433°N, 82.33335°W; depth 1–3 m, among red algae; 23 June 2023; K.N. White leg.; USNM 1703553 • 1 ♂, 2 ♀; Bocas del Toro, Swan Cay; 9.4536°N, 82.300033°W; depth 2 m, among red algae and coral rubble; 24 June 2023; K.N. White leg.; USNM 1703554.

###### Diagnosis.

Rostrum narrow, curved, distally acute. Gnathopods 1 and 2 propodus palm each with four spines and several setae. Epimeron 3 posterior margin regularly serrate. Uropod 3 inner ramus slightly shorter than outer ramus Telson cleft ½ of length, lobes not narrowing distally, apically rounded.

###### Distribution.

U.S.A.: Tampa Bay to the Florida Keys (Ortiz and Lalana 1996); Cuba: Gulf of Batabano (Ortiz and Lalana 1994), Cayo Mendoza (Ortiz and Lalana 1996); Columbia: south of Cartagena (Ortiz and Lemaitre 1994); Panama: Bocas del Toro (present study).

###### Ecology and remarks.

These amphipods are associated with algae and coral rubble at depths of 0–4 m. Panamanian specimens closely resemble previously described specimens. This species can be distinguished from the closely related *Tethygeneialongleyi* based on the narrow, distally acute rostrum, regularly serrate epimeron 3, and wide telson lobes. See LeCroy (2007) for discussion of these species.

**Figure 25.**
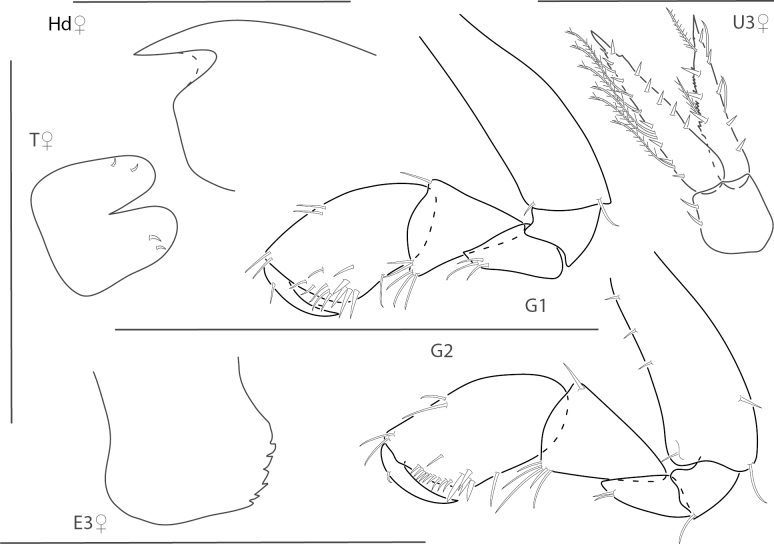
*Nasageneiabacescui*, female, 3.1 mm, head, telson, epimeron 3, uropod 3; male, 1.9 mm, gnathopods 1 and 2 medial. Scale bars: 0.5 mm.

##### ﻿Genus Tethygeneia

Taxon classificationAnimaliaAmphipodaPontogeneiidae

J.L. Barnard, 1972

B121C9A2-561E-5187-8ACF-F38ADE5EF00B

###### Diagnosis.

Rostrum reaching ~ 3/4 length of first article of antenna 1 peduncle, linguiform. Gnathopods 1 and 2 propodus relatively small, palmar margin lined with slender spines. Epimeron 3 posterior margin smooth or weakly serrate. Telson subrectangular, slightly longer than wide, apices of lobes rounded or subtruncate.

##### ﻿ Tethygeneia longleyi

Taxon classificationAnimaliaAmphipodaPontogeneiidae

(Shoemaker, 1933)

30448AB0-5C11-5736-8497-2A9D37AE8F86

Figs 26 , 30F

Pontogeneia longleyi Shoemaker, 1933: 253–254, figs 6, 7.
Tethygeneia longleyi : LeCroy 2007: 513, fig. 452.

###### Material examined.

Panama • 3–5 mm • 1 ♀; Bocas del Toro, Mangrove Inn; depth 1 m, among *Caulerpa*; 3 Aug 2005; M. Faust leg.; GCRL 6654 • 2 ♂, 1 ♀; Bocas del Toro, Drago; 9.418056°N, 82.3375°W; depth 2–3 m, among coral rubble, 9 Aug 2021; K.N. White leg.; USNM 1703555 • 3 ♂, 7 ♀, 1 juvenile; Bocas del Toro, Drago; 9.413433°N, 82.33335°W; depth 1–3 m; among *Halimeda* and *Dictyota*, 23 June 2023; K.N. White leg.; USNM 1703556 • 2 ♂, Bocas del Toro, Swan Cay; 9.4536°N, 82.300033°W; depth 2 m; among red algae, 24 June 2023; K.N. White leg.; USNM 1703557 • 2 ♂, 1 ♀, 3 juvenile; Bocas del Toro, Cayo Zapatilla 2; depth 0 m, buoy scraping; 29 June 2023; L. Hughes leg.; USNM 1703558.

###### Diagnosis.

Rostrum wide, curved, distally rounded. Gnathopods 1 and 2 propodus palm each with one to three spines and several setae. Epimeron 3 posterior margin smooth or slightly serrate. Uropod 3 inner ramus subequal in length to outer ramus Telson cleft 3/4 of length, lobes narrowing distally, apically rounded.

###### Distribution.

U.S.A.: Hutchinson Island to the Dry Tortugas, Florida (Shoemaker 1933; Camp et al. 1977; LeCroy 2007); Cuba: Archipélago Sabana-Camagüey (Ortiz and Lalana 1996); Venezuela: Maiguetia and Porlamar (Ruffo 1950); Brazil: Sao Paulo and Paraná (Wakabara and Serejo 1998); Panama: Bocas del Toro (present study).

###### Ecology and remarks.

These amphipods are associated with algae and sand at depths of 0–11 m. Panamanian specimens closely resemble previously described specimens in all aspects, and have the rounded telson apices as described by Shoemaker (1933) rather than the more subtruncate apices described by LeCroy (2007).

**Figure 26.**
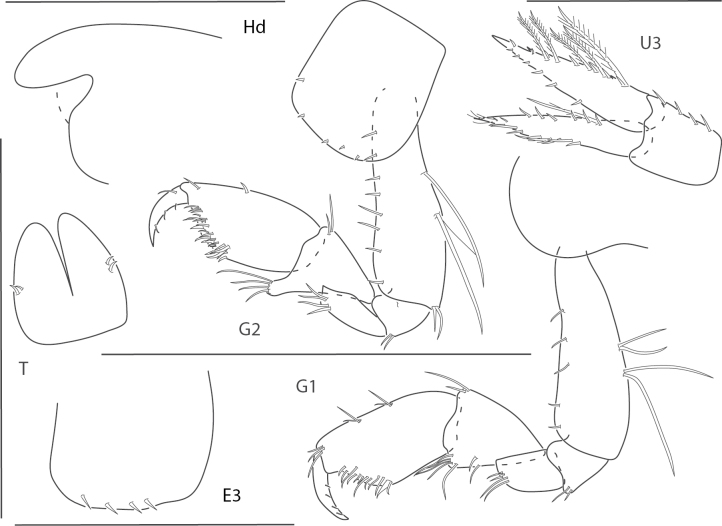
*Tethygeneialongleyi*, male, 2.1 mm, head, telson, epimeron 3, gnathopods 1 and 2 medial, uropod 3. Scale bars: 0.5 mm.

**Figure 27.**
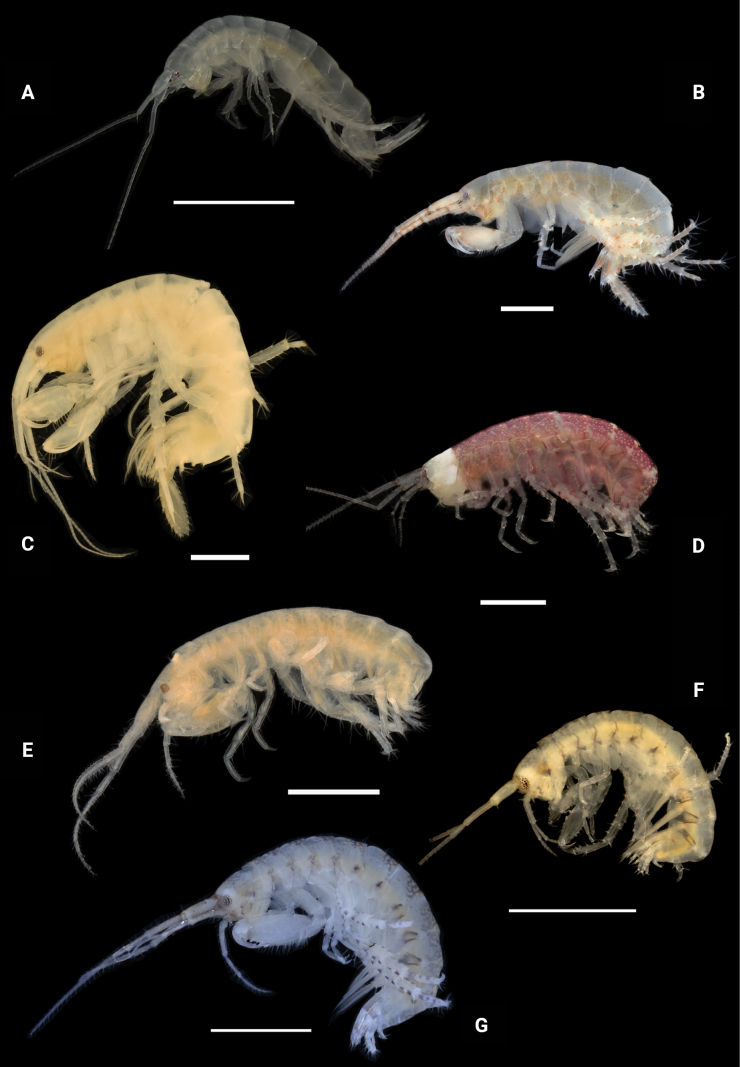
Photographs of live specimens **A***Dulzuraschoenerae***B***Ceraocussheardi***C***Ceradocusshoemakeri***D***Elasmopusbalkomanus***E**Elasmopuselieri (ethanol preserved specimen) **F***Elasmopuslevis***G***Elasmopuslongipropodus*. Scale bars: 1.0 mm.

**Figure 28.**
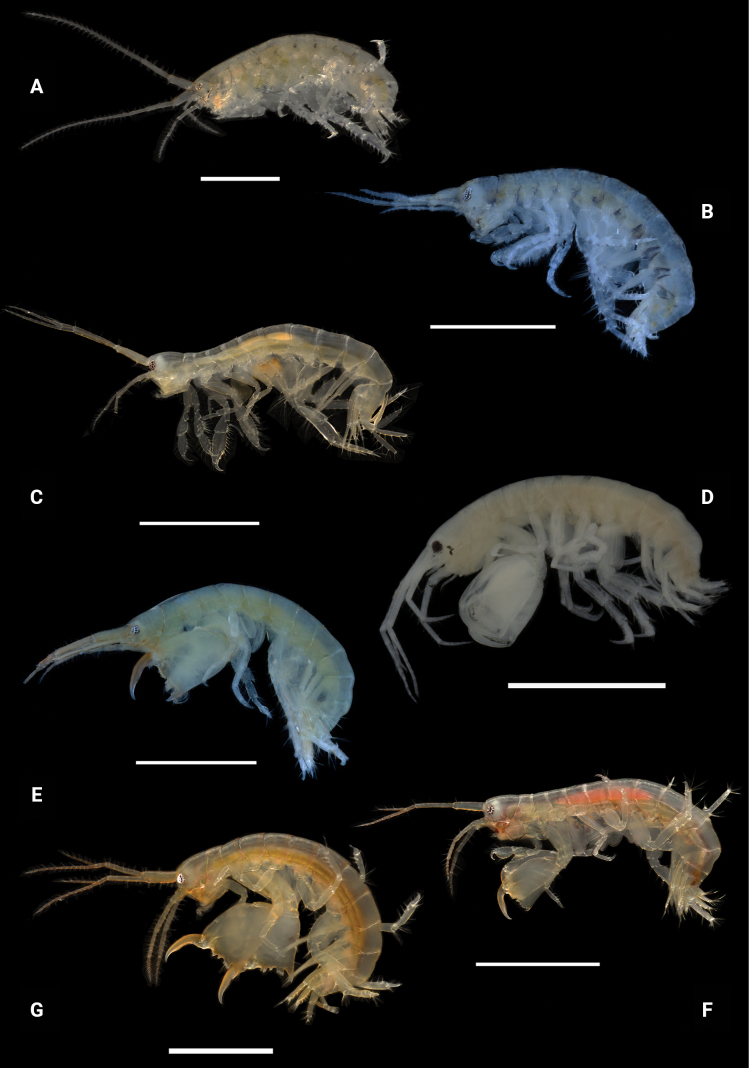
Photographs of live specimens unless noted **A***Elasmopuspocillimanus***B***Elasmopusthomasi***C***Meximaeradiffidentia***D***Quadrimaeraceres* (ethanol preserved specimen) **E***Quadrimaeracristianae***F***Quadrimaeramiranda***G***Quadrimaeraquadrimana*. Scale bars: 1.0 mm.

**Figure 29.**
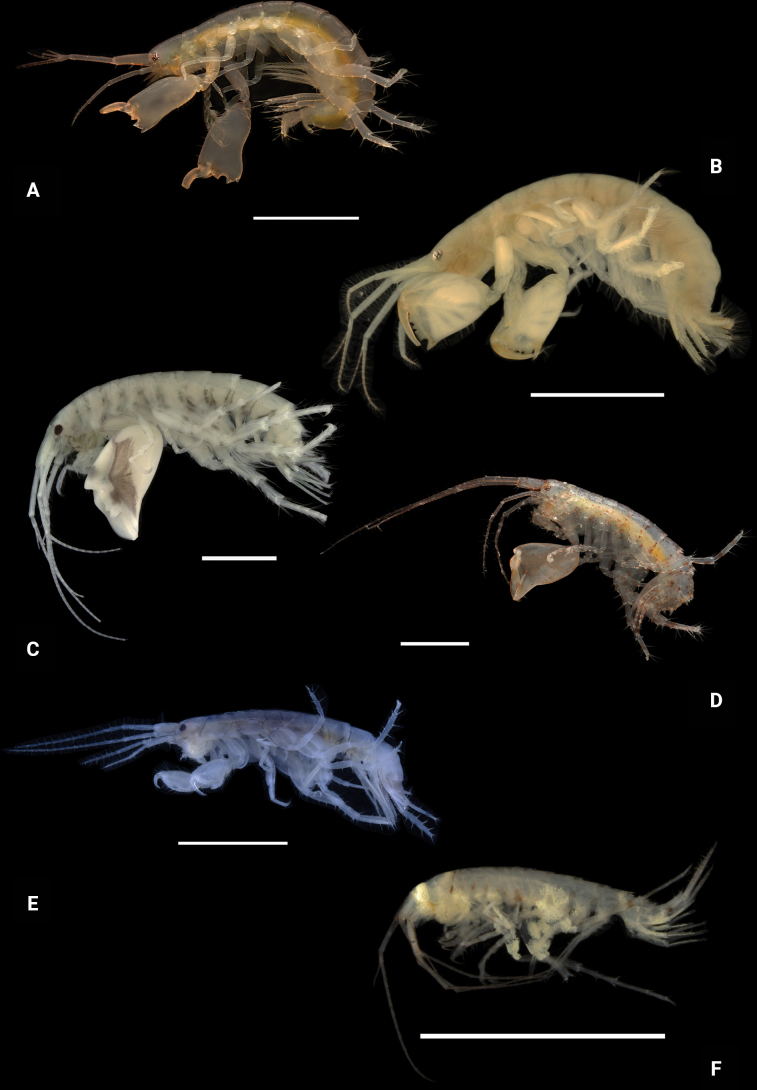
Photographs of live specimens unless noted **A***Quadrimaerasarae***B***Quadrimaerayemanjae***C***Dulichiellaanisochir* (ethanol preserved specimen) **D***Dulichiellalecroyae***E***Melitaplanaterga***F***Hornelliatequestae*. Scale bars: 1.0 mm.

**Figure 30.**
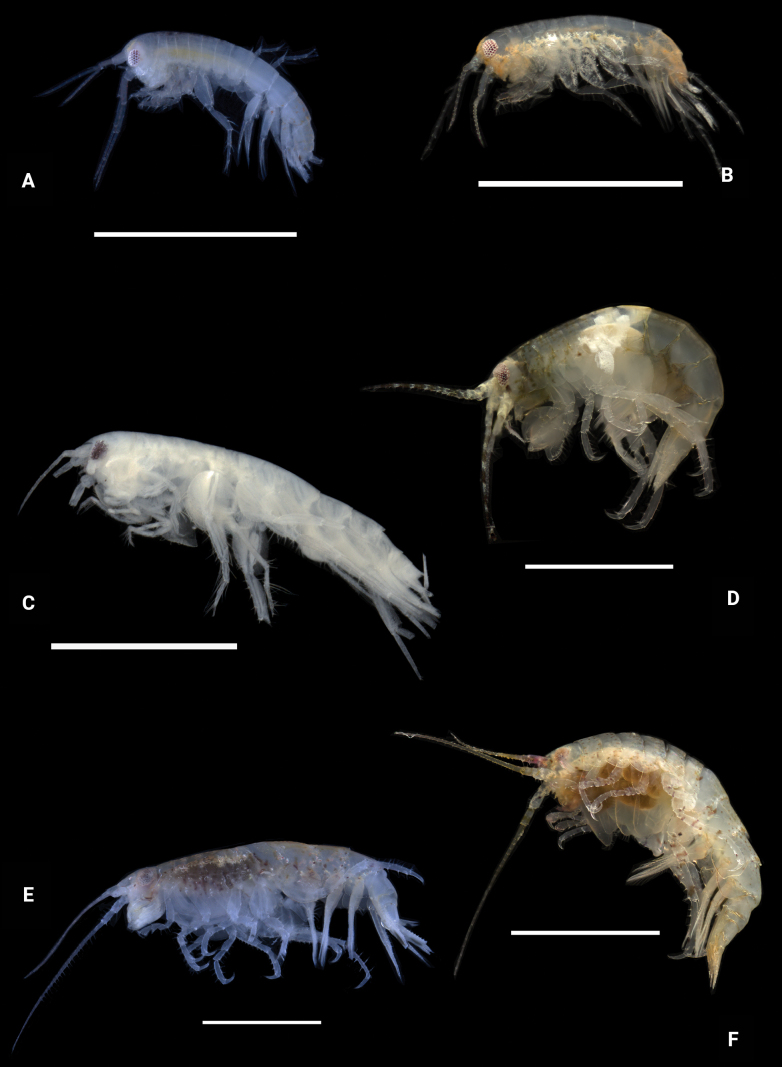
Photographs of live specimens unless noted **A***Gibberosusdevaneyi***B***Gibberosusmyersi***C***Resupinusspinicaudatus* (ethanol preserved specimen) **D***Eusiroidesyucatanensis***E***Nasageneiabacescui***F***Tethygeneialongleyi*. Scale bars: 1.0 mm.

### ﻿Identification Key to the Caribbean Hadziidira of Panama

**Table:** 

1	Gnathopod 2 simple; pereopod 7 elongate, distal articles extremely slender; uropod 3 rami broadly paddle-shaped (Fig. 21)	**2**
–	Gnathopod 2 subchelate; pereopod 7 not elongate, or if elongate, distal articles not unusually slender; uropod 3 rami not broadly paddle-shaped (Fig. 10)	**4**
2	Head rostrum short, ocular lobe with cusp; antenna 1 accessory flagellum 2-articulate; gnathopod 2 merus with large distal lobe; uropod 1 peduncle with interramal tooth (Fig. 21)	**3**
–	Head rostrum long, ocular lobe rounded; antenna 1 accessory flagellum 1-articulate; gnathopod 2 merus without distal lobe; uropod 1 peduncle without interramal tooth (Fig. 23)	***Resupinusspinicaudatus***
3	Head ocular lobe with subacute cusp; only pleosome segment 3 and urosome segment 2 with dorsal serrations; epimeron 3 smooth; uropod 3 rami continually lined with spines (Fig. 21)	***Gibberosusdevaneyi***
–	Head ocular lobe with acute cusp; pleosome segment 3 and urosome segments 1 and 2 with dorsal serrations; epimeron 3 serrate; uropod 3 rami with sparse marginal spines (Fig. 22)	***Gibberosusmyersi***
4	Gnathopod 2 similar in size to gnathopod 1, not strongly sexually dimorphic; uropod 1 peduncle without basofacial spine(s), outer ramus distinctly shorter than inner ramus	**5**
–	Gnathopod 2 larger than gnathopod 1, strongly sexually dimorphic; uropod 1 peduncle with basofacial spine(s), outer ramus subequal to or shorter than inner ramus	**8**
5	Antenna 1 accessory flagellum present (may be 1-articulate); telson lobes relatively narrow, apices acute or subacute (Fig. 24)	**6**
–	Antenna 1 lacking accessory flagellum; telson lobes relatively wide, apices rounded or subquadrate (Fig. 25)	**7**
6	Antenna 1 accessory flagellum 1-articulate; gnathopods 1 and 2 propodus palmar margin lined with peg-like robust setae; epimeron 3 posterior margin serrate, posteroventral corner not produced; telson distinctly longer than wide, apices of lobes subacute (Fig. 24)	***Eusiroidesyucatanensis***
–	Antenna 1 accessory flagellum 5-articulate; gnathopods 1 and 2 propodus palmar margin lined with slender setae; epimeron 3 posterior margin smooth, posteroventral corner produced; telson slightly longer than wide, apices of lobes bifid and acute (Fig. 20)	***Hornelliatequestae***
7	Rostrum narrow, distally acute; epimeron 3 regularly serrate; telson cleft ~ ½ of length, lobes not narrowing distally; uropod 3 inner ramus slightly shorter than outer ramus (Fig. 25)	***Nasageneiabacescui***
–	Rostrum wide, distally rounded; epimeron 3 smooth or with small serration; telson cleft ¾ of length, lobes narrowing distally; uropod 3 inner ramus subequal to outer ramus (Fig. 26)	***Tethygeneialongleyi***
8	Uropod 3 inner ramus minute (Fig. 1)	**9**
–	Uropod 3 rami subequal or slightly unequal in length (Figs 5, 7)	**12**
9	Gnathopod 2 of male, sides similar; posterodorsal margins of pleon segments 1–3 without serrations or teeth (Fig. 19)	**10**
–	Gnathopod 2 of male, sides dissimilar, significantly different in size, larger side chelate; posterodorsal margins of pleon segments 1–3 with serrations or teeth (Fig. 17)	**11**
10	Coxa 6 of female anterior lobe with lateral ridge at base of hook; uropod 3 outer ramus 1-articulate (Fig. 19)	***Melitaplanaterga***
–	Coxa 6 of female unmodified; uropod 3 outer ramus 2-articulate (Fig. 1)	***Dulzuraschoenerae***
11	Gnathopod 2 distolateral crown with four rounded or subacute spines, 4^th^ spine well developed, dactylus apically hooked, fitting into posterodistal corner; pereopods 6 and 7 carpus and propodus with bunches of long slender setae; epimeron 1 posteroventral corner subquadrate; epimeron 3 posterodistal margin smooth (Fig. 18)	***Dulichiellalecroyae***
–	Gnathopod 2 propodus distolateral crown with three rounded indistinct spines, dactylus apically blunt, overlapping posterodistal corner; pereopods 6 and 7 carpus and propodus without bunches of long slender setae; epimeron 1 posteroventral corner acute; epimeron 3 posterodistal margin serrate (Fig. 17)	***Dulichiellaanisochir***
12	Antenna 1 accessory flagellum 2- or 3-articulate; mandible, palp stout, article 3 falcate, with comb row of very short marginal setae; uropod 3 outer ramus < 3 × longer than wide (Fig. 5)	**13**
–	Antenna 1 accessory flagellum at least 4-articulate; mandible, palp slender, article 3 linear, without comb row of short marginal setae; uropod 3 outer ramus > 3 × longer than wide (Fig. 10)	**18**
13	Pereopod 7 basis posterior margin with long setae (Fig. 8)	**14**
–	Pereopod 7 basis posterior margin without long setae	**15**
14	Male gnathopod 2 propodus elongate, palm with three teeth; epimeron 3, posteroventral margin serrate (Fig. 7)	***Elasmopuslongipropodus***
–	Male gnathopod 2 propodus subovate, palm with large excavation; epimeron 3, posteroventral margin with single acute tooth (Fig. 8)	***Elasmopuspocillimanus***
15	Gnathopod 2 propodus palm with few setae; telson inner lobes apically rounded (Fig. 5)	**16**
–	Gnathopod 2 propodus palm densely setose; telson inner lobes apically acute (Fig. 4)	**17**
16	Male gnathopod 2 propodus palm concave with one triangular process; epimeron 3 posteroventral margin with small tooth; telson inner lobes subequal in length with outer lobes (Fig. 5)	***Elasmopuselieri***
–	Male gnathopod 2 propodus palm with 3 processes and 2 notches; epimeron 3 posterior margin serrate; telson inner lobes longer than outer lobes (Fig. 9)	***Elasmopusthomasi***
17	Pereopod 5 basis posterior margin concave; telson inner lobes shorter than outer lobes (Fig. 4)	***Elasmopusbalkomanus***
–	Pereopod 5 basis posterior margin evenly convex; telson inner lobes subequal to outer lobes (Fig. 6)	***Elasmopuslevis***
18	Mandible palp article 1 with small distal tooth; maxilla 2 inner plate with dense oblique row of facial setae; uropod 3 rami broad, foliaceous, tips subacute (Fig. 2)	**19**
–	Mandible palp article 1 without small distal tooth (Fig. 10); maxilla 2 inner plate without dense oblique row of facial setae (Fig. 16); uropod 3 rami slender, tips subtruncate (Fig. 10)	**20**
19	Male gnathopod 2 left and right sides dissimilar; pleon segments 1–3 posterodorsal margins without strong teeth or serrations; urosome segments 1 and 2 posterodorsal margins each with single tooth (Fig. 3)	***Ceradocusshoemakeri***
–	Male gnathopod 2 left and right sides similar; pleon segments 1–3, posterodorsal margins with many strong teeth or serrations; urosome segments 1 and 2 posterodorsal margins with many strong teeth (Fig. 2)	***Ceradocussheardi***
20	Eyes oval; gnathopod 1 carpus subequal to propodus, without dorsal excavation; gnathopod 2 propodus palm oblique; pereopods with simple dactyli; pereopod 7 basis slimmer than long, without posterodistal lobe (Fig. 10)	***Meximaeradiffidentia***
–	Eyes round; gnathopod 1 carpus longer than propodus, with dorso-distal excavation; gnathopod 2 propodus palm with right angle; pereopods with bifid dactyli; pereopod 7 basis as slim as long, with posterodistal lobe (Fig. 11)	**21**
21	Male gnathopod 2 propodus palm with deep excavations, palmar angle defined by elongate process below deep U-shaped notch (Fig. 14)	**22**
–	Male gnathopod 2 propodus palm with shallow excavations, palmar angle defined by short process below shallow V-shaped notch (Fig. 15)	**24**
22	Gnathopod 2 dactyl inner margin smooth in males and females; telson lobes apically truncate (Fig. 14)	**23**
–	Gnathopod 2 dactyl inner margin inflated in males and females; telson inner lobes inner corner acutely produced (Fig. 12)	***Quadrimaeracristianae***
23	Gnathopod 1 propodus palm with short-to-medium setae; gnathopod 2 propodus palm with two large U-shaped excavations; telson with lateral plumose setae (Fig. 14)	***Quadrimaeraquadrimana***
–	Gnathopod 1 propodus palm with long setae; gnathopod 2 propodus palm with one large and two small excavations; telson without lateral setae (Fig. 16)	***Quadrimaerayemanjae***
24	Gnathopod 2 propodus palm transverse with central U-shaped concavity and subquadrate/quadrate processes, dactylus strong, medially expanded (Fig. 15)	***Quadrimaerasarae***
–	Gnathopod 2 propodus palm transverse with smaller, deeper concavity and broad processes; dactylus not strong (Fig. 11)	**25**
25	Gnathopod 2 dactylus inner margin inflated with median point (Fig. 13)	***Quadrimaeramiranda***
–	Gnathopod 2 dactylus inner margin inflated without median point (Fig. 11)	***Quadrimaeraceres***

## ﻿Discussion

The results of this study represent range extensions for 26 species of hadziidirid amphipods to include Caribbean waters of Panama. Several species have a distribution pattern spanning the eastern Pacific and western Caribbean (*Elasmopuspocillimanus*, *Meximaeradiffidentia*, *Quadrimaeraquadrimana*, *Gibberosusdevaneyi*, and *Gibberosusmyersi*). Without examining material from every collection, it is impossible to be sure the species in the literature were identified correctly or if the specimens may represent a different species. Assuming proper identification, these distribution patterns may suggest that the species were established more than 3 mya, before the isthmus of Panama closed.

Several hadzidiiran species demonstrate variation in key characters or are identified based on males only. Several *Elasmopus* species are differentiated based on epimeron 3 serration patterns and the apices of telson lobes, both of which can vary among individual specimens. As discussed by LeCroy (2007)*Tethygeneia* and *Pontogeneia* show variation in epimeron 3 serration, which is a key character for identification of these species. Our identification key allows identification despite variation of both males and females of the Caribbean Hadziidira of Panama.

## Supplementary Material

XML Treatment for Dulzura

XML Treatment for Dulzura schoenerae

XML Treatment for Ceradocus

XML Treatment for Ceradocus sheardi

XML Treatment for Ceradocus shoemakeri

XML Treatment for Elasmopus

XML Treatment for Elasmopus balkomanus

XML Treatment for Elasmopus elieri

XML Treatment for Elasmopus levis

XML Treatment for Elasmopus longipropodus

XML Treatment for Elasmopus pocillimanus

XML Treatment for Elasmopus thomasi

XML Treatment for Meximaera

XML Treatment for Meximaera diffidentia

XML Treatment for Quadrimaera

XML Treatment for Quadrimaera ceres

XML Treatment for Quadrimaera cristianae

XML Treatment for Quadrimaera miranda

XML Treatment for Quadrimaera quadrimana

XML Treatment for Quadrimaera sarae

XML Treatment for Quadrimaera yemanjae

XML Treatment for Dulichiella

XML Treatment for Dulichiella anisochir

XML Treatment for Dulichiella lecroyae

XML Treatment for Melita

XML Treatment for ﻿Melita planaterga

XML Treatment for Hornellia

XML Treatment for Hornellia tequestae

XML Treatment for Gibberosus

XML Treatment for Gibberosus devaneyi

XML Treatment for Gibberosus myersi

XML Treatment for Resupinus

XML Treatment for Resupinus spinicaudatus

XML Treatment for Eusiroides

XML Treatment for Eusiroides yucatanensis

XML Treatment for Nasageneia

XML Treatment for Nasageneia bacescui

XML Treatment for Tethygeneia

XML Treatment for Tethygeneia longleyi

## ﻿Additional information

### Author ORCIDs

Kristine N. White https://orcid.org/0000-0002-5203-1656

Sally J. Sir https://orcid.org/0000-0002-1270-1192

### Data availability

All of the data that support the findings of this study are available in the main text or Supplementary Information.

## Supplementary materials

Supplementary material 1 Locality table Data type xlsx https://binary.pensoft.net/file/1005013This dataset is made available under the Open Database License (http://opendatacommons.org/licenses/odbl/1.0/). The Open Database License (ODbL) is a license agreement intended to allow users to freely share, modify, and use this Dataset while maintaining this same freedom for others, provided that the original source and author(s) are credited.Kristine N. White, Sally J. Sir
